# PSMD14‐Mediated LDHA Deubiquitination Upregulates ACLY Expression via H3K18 Lactylation to Promote Lipid Synthesis and Pancreatic Cancer Progression

**DOI:** 10.1002/advs.202505762

**Published:** 2025-10-06

**Authors:** Ri‐Shang Lu, Li‐Kun Ren, Xiao‐Bin Fei, Song‐Bai Liu, Yong‐Jia Gao, Jun‐Yi Hou, Chi Wang, Peng Liu, Chang‐Hao Zhu, Xing Wang, Yao‐Zhen Pan

**Affiliations:** ^1^ School of Clinical Medicine Guizhou Medical University Guiyang 550004 China; ^2^ Department of Hepatobiliary Surgery Affiliated Cancer Hospital of Guizhou Medical University Guizhou Medical University Guiyang 550001 China; ^3^ Department of Hepatobiliary Surgery Zhejiang Provincial People's Hospital Hangzhou Medical University Hangzhou 310014 China; ^4^ Department of Hepatobiliary Surgery Baiyun Hospital Guizhou Medical University Guiyang 550001 China; ^5^ Department of Hepatobiliary Surgery Affiliated Hospital of Guizhou Medical University Guiyang 550001 China

**Keywords:** Deubiquitination, histone lactylation, LDHA, lipid metabolism, pancreatic cancer

## Abstract

Aberrant lipid metabolism is intimately linked to tumor progression. As a pivotal post‐translational modification, ubiquitination regulates diverse oncogenic processes. However, the interplay between ubiquitination and lipid metabolic dysregulation in pancreatic cancer (PC), along with its underlying molecular mechanisms, remains poorly understood. Here, it is demonstrated that glycolytic enzyme lactate dehydrogenase A (LDHA) potentiates lipid biosynthesis under the regulation of deubiquitinases. Specifically, PSMD14 directly binds and stabilizes LDHA through its deubiquitinase activity, resulting in intracellular lactate accumulation. Elevated lactate levels enhance histone lactylation marks, which transcriptionally activate ATP citrate lyase (ACLY) to promote malignant progression via fatty acid synthesis pathway activation. This study reveals a previously unrecognized role of PSMD14‐derived lactate in mediating histone lactylation‐coupled lipid deposition and tumor progression. Therapeutic co‐targeting of PSMD14 and glycolytic lactylation significantly suppresses tumor growth in patient‐derived xenograft models, suggesting a promising combinatorial strategy for pancreatic cancer treatment.

## Graphical abstract

This study investigates the expression and functional role of the deubiquitinase PSMD14 in PC, with a focus on the mechanism by which it promotes lactate accumulation–induced histone lactylation, thereby driving aberrant lipid metabolism. These findings establish that dual targeting of PSMD14 and the glycolysis–lactylation signaling axis may represent a promising therapeutic strategy for PC.

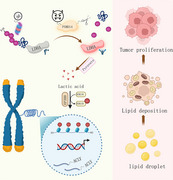

## 1 Introduction

Pancreatic cancer (PC) is a highly aggressive malignant tumor that threatens human health, with its incidence increasing at an annual rate of ≈1%. It is estimated to become the second leading cause of cancer‐related deaths worldwide by 2030.^[^ ^1^ ^]^ Owing to the insidious nature of early symptoms and the lack of effective screening methods, many patients are diagnosed at advanced stages or with remote metastasis, thereby abrogating the possibility for a curative operation.^[^ ^2^ ^]^ Despite recent advancements in immunotherapy, the survival rate of patients with PC has not significantly increased, and its mortality rate remains one of the highest among malignant tumors.^[^ ^3^ ^]^ Complex metabolic reprogramming, including abnormal glycolysis activation, glutamine reliance, and lipid metabolism imbalance, is involved in the pathogenesis of PC. These adaptive changes collectively drive rapid tumor progression and treatment resistance.^[^ ^4^ ^]^ Therefore, in‐depth exploration of key regulatory mechanisms involved in metabolic reprogramming is essential for improving the treatment of PC.

Extensive research has shown that metabolic reprogramming is one of the core biological characteristics of malignant progression and treatment resistance in PC, driving tumor evolution by altering the energy supply and reshaping biosynthetic networks.^[^ ^5^ ^]^ Abnormal lipid metabolism is particularly prominent in PC.^[^ ^6^ ^]^ Cancer cells require a large supply of energy to support their rapid proliferation, and lipids are a vital energy source that promotes cancer cell proliferation.^[^ ^7^ ^]^ Lipid molecules provide essential energy carriers (ATP) and biomembrane components for the rapid proliferation of cancer cells;^[^ ^8^ ^]^ moreover, lipid metabolism‐derived signaling molecules (such as lysophosphatidic acid and prostaglandins) can further lead to immune cell dysfunction and induce chemotherapy resistance.^[^ ^9^ ^]^ Given the highly malignant pathological features of PC and the currently employed clinical treatment challenges, systematically elucidating the key molecular regulatory mechanisms of tumor metabolic reprogramming and identifying potential drug intervention targets holds significant scientific value for overcoming existing treatment bottlenecks and improving patient clinical outcomes.

The ubiquitin–proteasome system (UPS) plays an essential role in regulating protein stability, and abnormal UPS function can disrupt intracellular homeostasis and support tumorigenesis.^[^ ^10^ ^]^ Emerging evidence suggests that ubiquitination is an important process in cellular lipid metabolism.^[^ ^11^ ^]^ Deubiquitinating enzymes (DUBs), as core regulators of protein homeostasis, reverse the ubiquitination of substrate proteins to regulate their function and stability, thereby playing significant roles in key biological events such as cell signaling, DNA damage repair, and metabolic reprogramming.^[^ ^12^ ^]^ For example, Ubiquitin‐Specific Protease 7 (USP7) drives tumorigenesis and de novo lipogenesis in head and neck squamous cell carcinoma by stabilizing Ras‐GTPase Activating Protein SH3 Domain‐Binding Protein 2 (G3BP2);^[^ ^12d^ ^]^ additionally, Ubiquitin Specific Peptidase 22 (USP22) can influence lipid accumulation in liver cancer cells by regulating key lipid metabolism factors such as Peroxisome Proliferator‐Activated Receptor Gamma (PPARγ).^[^ ^13^ ^]^ PSMD14 (POH1), a member of the JAMM superfamily, is a deubiquitinating enzyme.^[^ ^14^ ^]^ As an essential subunit of the 26S proteasome, PSMD14 not only participates in critical cellular processes such as DNA damage repair, genomic transcription regulation, tumor chemotherapy resistance, and cellular senescence.^[^ ^15^ ^]^ but has also been shown to stabilize MYC through deubiquitination, driving acinar‐ductal metaplasia, a necessary process for the initiation of pancreatic ductal adenocarcinoma.^[^ ^16^ ^]^ Nevertheless, the regulatory role of PSMD14 in lipid metabolic reprogramming in PC and the involved molecular mechanisms remain unknown, and this research gap urgently requires further exploration.

In the present study, we first revealed the critical role and clinical significance of the deubiquitinating enzyme PSMD14 in mediating lipid metabolic reprogramming in PC. Analysis of clinical samples revealed that PSMD14 is abnormally highly expressed in PC and that its high expression level is significantly associated with poor patient prognosis. Mechanistically, PSMD14 stabilizes lactate dehydrogenase (LDHA) through deubiquitination, leading to abnormally elevated lactate levels in tumor cells, which in turn drives increased lactylation at the H3K18 site (H3K18la). These epigenetic modifications activate the transcription of ATP citrate lyase (ACLY) through chromatin remodeling, ultimately promoting malignant tumor progression by enhancing lipid biosynthesis metabolism. These results support the role of PSMD14 in regulating tumor metabolism and epigenetics in PC and indicate that PSMD14 may be a possible therapeutic target for PC.

## 2 Results

### 2.1 PSMD14 Promotes Lipid Deposition and Fatty Acid Synthesis in PC Cells

**Figure 1:**
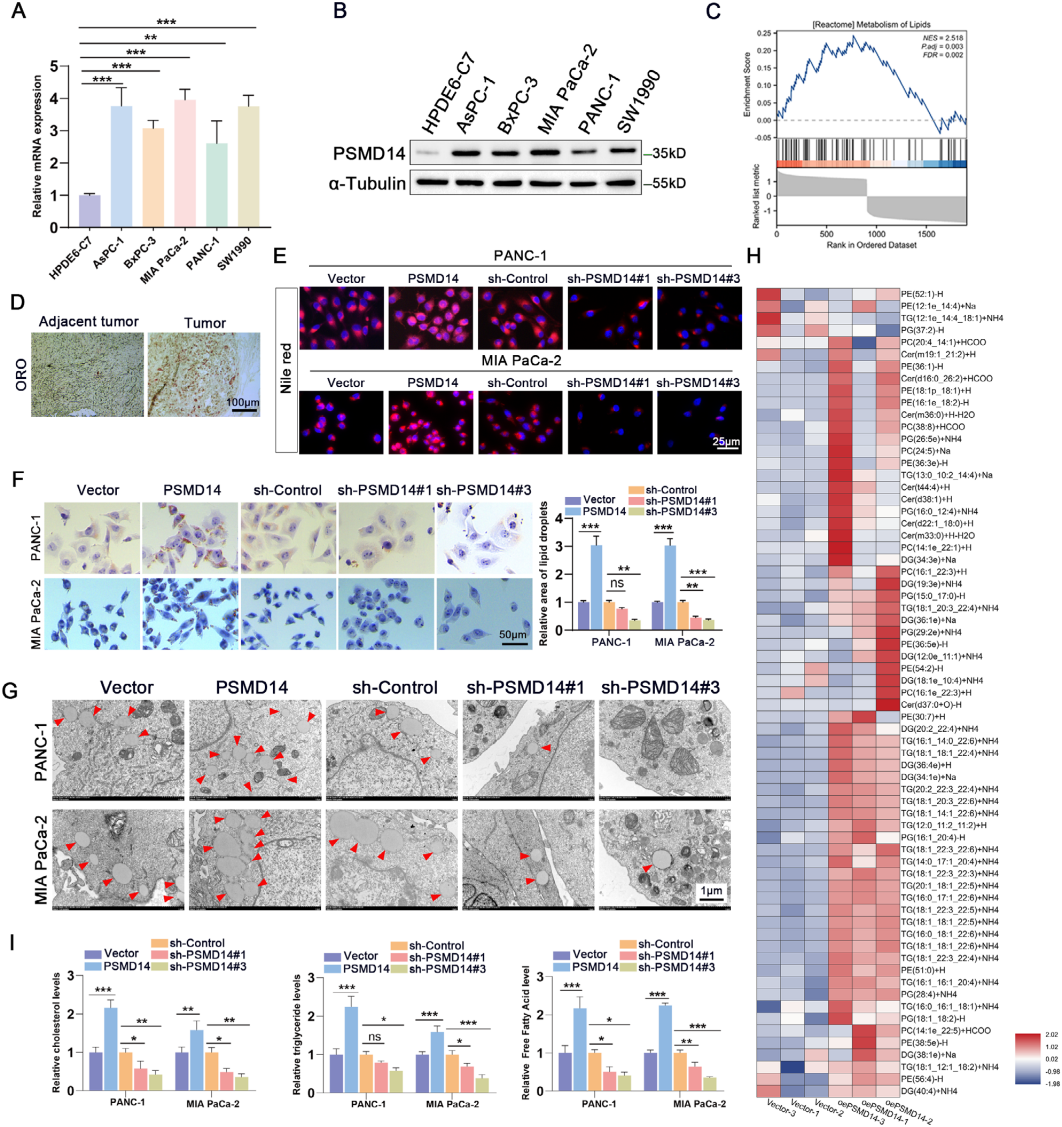
PSMD14 promotes fatty acid synthesis and lipid deposition in pancreatic cancer cells. A) The mRNA level of the PSMD14 gene in human normal pancreatic ductal epithelial cells HPDE6‐C7 and pancreatic cancer cell lines was detected by qRT‐PCR; *n* = 3 biologically independent samples. B) Western blot analysis of PSMD14 in human normal pancreatic ductal epithelial cells HPDE6‐C7 and pancreatic cancer cell lines; *n* = 3 biologically independent samples. C) RNA‐seq analysis was performed after overexpression of PSMD14 in PANC‐1 cells, and GSEA showed that PSMD14 was involved in regulating the signaling pathways of lipid metabolism in PC (FDR = 0.002, *P* = 0.003). D) Lipid accumulation in PC tissues was detected by Oil Red O (ORO) staining; n = 30 biologically independent samples. E) PC cells were transfected with PSMD14 expression vector (oePSMD14), 2 separate shRNAs targeting different regions of the PSMD14 gene (shPSMD14‐2#, shPSMD14‐3#) or their corresponding control vectors, followed by Nile Red staining to detect lipid deposition in cells. *n* = 3 biologically independent samples. F) Representative images and statistical analysis of lipid droplets in PC cells with different PSMD14 expression levels detected by Oil Red O staining; *n* = 3 biologically independent samples. G) Lipid droplets in PC cells were detected by electron microscopy; *n* = 3 biologically independent samples. H) Heatmap showing changes in lipid components in PANC‐1 cells overexpressing PSMD14; *n* = 3 biologically independent samples. I) The contents of triglycerides (TG), free fatty acids, and cholesterol in PC cells were determined as quantitative indicators of lipid deposition; *n* = 3 biologically independent samples. Data are presented as mean ± standard deviation (SD). *P < 0.05, **P < 0.01, ***P < 0.001. *P*‐values were determined by unpaired two‐tailed Student's *t*‐test (F,I) and one‐way ANOVA (A,F,I).

Disorder of the UPS plays a vital role in lipid metabolic reprogramming in tumor cells.^[^ ^11^ ^]^ To identify the link between deubiquitinating enzymes and lipid metabolism in PC, we performed a weighted gene coexpression network analysis (WGCNA) using data obtained from a deubiquitinating enzyme database (Figure S1A–F, Supporting Information). Notably, the deubiquitinating enzyme PSMD14 was closely associated with PC (Figure S2A–E, Supporting Information). Analysis of data from the Cancer Genome Atlas (TCGA) revealed that PSMD14 was strongly upregulated in PC tissues, and its high expression was correlated with poor patient prognosis (Figure S3A,B, Supporting Information). To further research the relationship between PC and PSMD14 expression, PSMD14 expression was first validated in cell lines and PC tissues using Western blotting and qRT‐PCR. The outcomes demonstrated that PSMD14 expression was upregulated in PC tissues compared with paired adjacent normal tissues (Figure S3C,D, Supporting Information). Similarly, PSMD14 expression was markedly greater in cultured PC cell lines (AsPC‐1, BxPC‐3, MIA PaCa‐2, PANC‐1, and SW 1990) than in human pancreatic ductal epithelial cells (HPDE6‐C7) (**Figure** **1**A,B). The IHC scores for PSMD14 were considerably higher in PC tissues than in normal tissues (Figure S3E,F, Supporting Information) and correlated with clinical stage, distant metastasis, neurological invasion, and tumor differentiation (Table S1, Supporting Information). Cox proportional hazards regression models identified PSMD14 IHC scores as an independent risk factor for PC prognosis (Table S2, Supporting Information). Kaplan–Meier survival analysis revealed significantly shorter overall survival in patients with elevated PSMD14 expression (Figure S3G, Supporting Information), emphasizing the relationship between PSMD14 expression and PC patient prognosis.

These results indicate that PSMD14 plays an oncogenic role in promoting PC progression, but researchers have not clearly determined whether it regulates PC progression by influencing lipid metabolism. To clarify the causal relationship between PSMD14 upregulation and lipid accumulation, we constructed stable cell lines with PSMD14 overexpression or knockdown (Figure S3H–K, Supporting Information). Gene set enrichment analysis (GSEA) of transcriptomic data from PSMD14‐overexpressing PC cells revealed a strong association between PSMD14 and lipid metabolism processes (Figure 1C). Oil Red O staining revealed greater lipid deposition in PC tissues than in contiguous normal tissues (Figure 1D). Subsequent experiments in PANC‐1 and MIA PaCa‐2 cells with PSMD14 overexpression or silencing further supported these observations. PSMD14 overexpression significantly increased lipid accumulation in PC cells, whereas PSMD14 knockdown reduced lipid accumulation, as demonstrated by Oil Red O and Nile Red staining (Figure 1E,F). Transmission Electron Microscopy (TEM) revealed that PSMD14 overexpression led to an increase in the number of lipid droplets, whereas PSMD14 downregulation was accompanied by a decrease in the number of lipid droplets (Figure 1G). To further validate these findings, we performed global lipidomic profiling via mass spectrometry to systematically compare lipid composition between PSMD14‐overexpressing pancreatic cancer (PC) cells and empty vector controls. Heatmap analysis revealed that among the 67 most differentially abundant lipid species, PSMD14‐overexpressing PANC‐1 cells displayed significant upregulation across multiple lipid classes (Figure 1H).

Subsequently, we measured the levels of intracellular triglycerides (TG), free fatty acids, and cholesterol in pancreatic cancer cells to assess the effect of PSMD14 on lipid metabolism in pancreatic cancer cells. The results revealed that PANC‐1 and MIA PaCa‐2 cells with stable PSMD14 overexpression showed significantly higher levels of TG, free fatty acids, and cholesterol compared to cells with PSMD14 knockdown (Figure 1I). In summary, these findings support a critical role of PSMD14 in regulating lipid metabolism in PC cells.

To elucidate PSMD14's role in lipid metabolism regulation, we first examined its association with key fatty acid synthesis enzymes using TCGA‐PAAD transcriptomic data. Bioinformatics analysis demonstrated significant positive correlations between PSMD14 expression and both ATP‐citrate lyase and acetyl‐CoA carboxylase 1 (Figure S4A,B, Supporting Information). Subsequently, multiplex immunofluorescence analysis of pancreatic cancer tissues demonstrated co‐upregulation of ACLY and ACC1 proteins in PSMD14‐high tumor regions (Figure S4C, Supporting Information). These results further indicate that PSMD14 plays a regulatory role in lipid metabolism.

### 2.2 PSMD14 Promotes PC Cell Proliferation In Vitro and In Vivo

To evaluate the biological functions of PSMD14 in PC development, we examined the effects of PSMD14 on the proliferation of MIA PaCa‐2 and PANC‐1 cells. EdU and CCK‐8 assays revealed that reduced PSMD14 expression inhibited the proliferation of MIA PaCa‐2 cells and PANC‐1 cells, while increased PSMD14 expression significantly promoted PC cell proliferation (Figure S5A,B, Supporting Information). Additionally, flow cytometry analysis revealed that PSMD14 depletion led to a significant increase in the proportion of MIA PaCa‐2 cells and PANC‐1 cells in the G0/G1 phase, whereas PSMD14 overexpression resulted in a significant decrease in the proportion of cells in the G0/G1 phase (Figure S5C,D, Supporting Information).

To further investigate the oncogenic role of PSMD14 in vivo, we subcutaneously injected stable PSMD14‐overexpressing and PSMD14‐knockdown PANC‐1 cells into nude mice to establish a xenograft tumor model. In vivo studies demonstrated that PSMD14 silencing significantly inhibited tumor formation, whereas PSMD14 overexpression accelerated subcutaneous tumor growth, as shown by weight and tumor volume measurements (Figure S5E, Supporting Information). Subsequent immunohistochemical staining of the xenograft tumors revealed that the Ki67 and PCNA expression levels were significantly greater in the PSMD14‐overexpressing tumors than in the control tumors and significantly lower in the PSMD14‐knockdown tumors (Figure S5F, Supporting Information).

### 2.3 PSMD14 Interacts with LDHA

**Figure 2:**
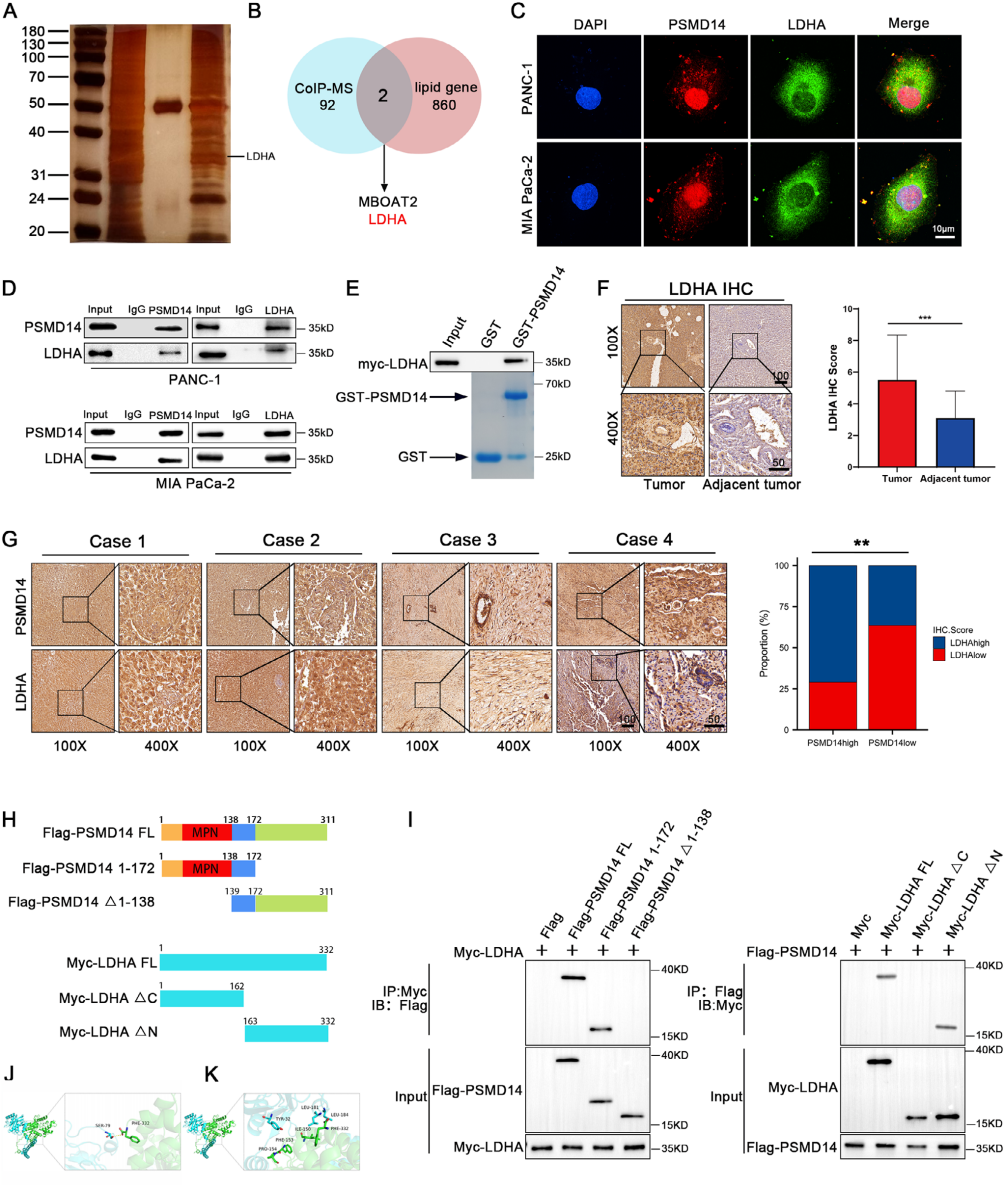
PSMD14 interacts with LDHA. A) Silver staining image before co‐immunoprecipitation combined with mass spectrometry (CoIP‐MS) analysis. B) Venn diagram showing the results of cross‐analysis between mass spectrometry‐based proteomics data and core gene sets of fatty acid metabolism. C) Representative images of immunofluorescence co‐localization staining for PSMD14 and LDHA in PANC‐1 and MIA PaCa‐2 cells; *n* = 3 biologically independent samples. D) CoIP combined with Western blot to detect the interaction between endogenous PSMD14 and LDHA in PANC‐1 and MIA PaCa‐2 cells; *n* = 3 biologically independent samples. E) GST‐pull down assay to detect the interaction between PSMD14 and LDHA; *n* = 3 biologically independent samples. F) IHC to detect the expression of LDHA in PC and corresponding adjacent tissues; *n* = 60 biologically independent samples. G) Representative images of IHC staining for PSMD14 and LDHA in pancreatic cancer tissues; *n* = 60 biologically independent samples. H) Domain structures and deletion mutants of PSMD14 and LDHA used in the study. I) Immunoprecipitation assay showing that PSMD14 interacts with LDHA through its UBD domain (1‐172), which contains the MPN domain (58‐138), while the C‐terminus of LDHA is physically interacting with PSMD14; *n* = 3 biologically independent samples. J) Diagram illustrating the interaction between PSMD14 and LDHA proteins, along with the names of amino acid residues involved in hydrogen bond formation. The blue segment represents the PSMD14 protein, and the green segment represents the LDHA protein. Hydrogen bonds are denoted by yellow dashed lines. K) Interaction between PSMD14 and LDHA proteins, including the names of amino acid residues participating in hydrophobic interactions. The blue segment corresponds to the PSMD14 protein, and the green segment corresponds to the LDHA protein. Data are presented as mean ± standard deviation (SD). *P < 0.05, **P < 0.01, ***P < 0.001. *P*‐values were determined by paired two‐tailed Student's *t‐*test (F [right]) and Chi‐square test (G [right]).

To explore the mechanism by which PSMD14, a deubiquitinating enzyme, regulates lipid synthesis in PC cells, we first identified 94 potential protein‐binding partners of PSMD14 through Co‐IP followed by mass spectrometry (**Figure** **2**A). By comparing these potential targets with lipid metabolism‐related genes, we discovered that LDHA might be an effector molecule through which PSMD14 regulates lipid synthesis (Figure 2B). To characterize the spatial distribution of LDHA and PSMD14, we performed subcellular localization analysis in pancreatic carcinoma cells. Immunofluorescence colocalization revealed that LDHA and PSMD14 colocalized in both the nuclei and cytoplasms of PC cells (Figure 2C). Co‐IP experiments confirmed that endogenous PSMD14 coprecipitated with endogenous LDHA (Figure 2D). Subsequently, GST pull‐down assays demonstrated that PSMD14 and LDHA could interact in vitro (Figure 2E). Proximity ligation assay (PLA) further confirmed the direct interaction between PSMD14 and LDHA in cells (Figure S6A, Supporting Information). IHC experiments revealed that LDHA expression was greater in PC tissues than in adjacent normal tissues. A positive correlation between the IHC staining scores of LDHA and PSMD14 in PC tissues was observed (Figure 2F,G).

The PSMD14 protein consists of an N‐terminal putative ubiquitin‐binding domain (UBD), an MPN domain (the catalytic domain for deubiquitination), and a C‐terminal domain (Figure 2H). To analyze the interaction between LDHA and PSMD14, we created deletion constructs. The results indicated that the UBD of PSMD14 is essential for its interaction with LDHA, while the interaction between LDHA and PSMD14 is mediated by the C‐terminal domain of LDHA (Figure 2I). Additionally, computational modeling was used to further analyze the interaction mechanism between PSMD14 and LDHA. Molecular docking results showed that the protein–protein docking score between PSMD14 and LDHA was −278.13, and hydrogen bonding interactions were formed between SER79 in PSMD14 and PHE332 in LDHA, with a hydrogen bond distance of 3.2 Å. The residues TYR32, LEU181, and LEU184 in the PSMD14 protein form hydrophobic interactions with the residues PRO154, PHE153, ILE150, and PHE332 in the LDHA protein (Figure 2J,K). Considering the established role of PSMD14 as a deubiquitinating enzyme and its documented interaction with LDHA, we hypothesize that PSMD14 may participate in modulating the post‐translational regulation of LDHA.

### 2.4 PSMD14 Regulates LDHA Protein Stability

**Figure 3:**
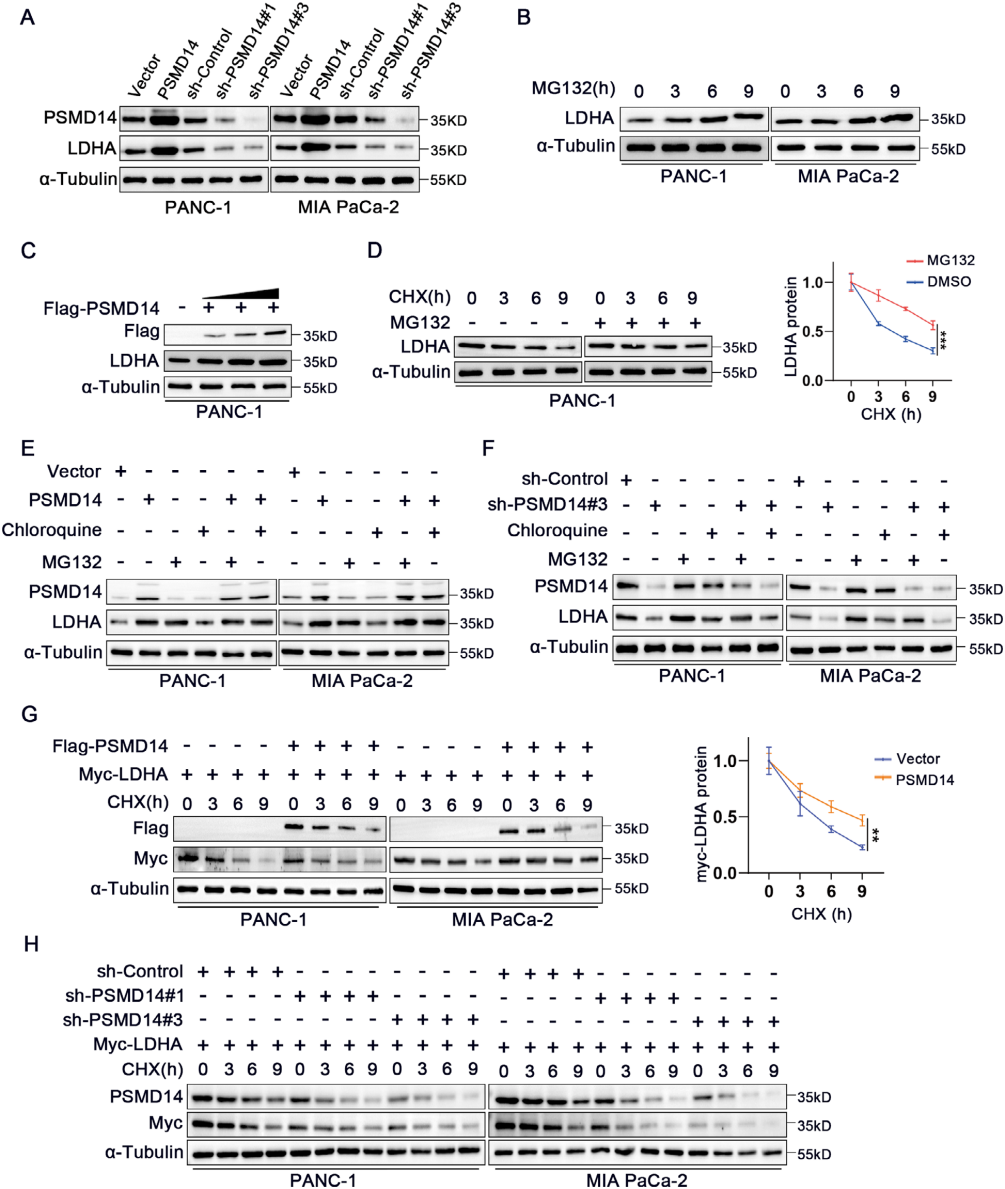
PSMD14 regulates the stability of the LDHA protein. A) Western blot was used to detect the level of LDHA in PANC‐1 and MIA PaCa‐2 cells after PSMD14 knockdown or overexpression; *n* = 3 biologically independent samples. B) Western blot results showing the changes in LDHA expression level with the prolongation of MG132 (20 µm) treatment time; *n* = 3 biologically independent samples. C) After gradient transfection of the Flag‐PSMD14 plasmid into PANC‐1 cells, the protein level of LDHA was detected by Western blot; *n* = 3 biologically independent samples. D) The half‐life of LDHA protein was detected by Western blot after treating PANC‐1 cells with the proteasome inhibitor MG132 (20 µm); *n* = 3 biologically independent samples. E,F) After treating PANC‐1 cells with PSMD14 overexpression or knockdown with MG132 (20 µm) for 8 h, the level of LDHA protein was detected by Western blot analysis; *n* = 3 biologically independent samples. G,H) After treating cells with the protein synthesis inhibitor CHX (100 µg mL^−1^) for the specified time, the degradation of LDHA protein at different time points was detected by Western blot analysis; *n* = 3 biologically independent samples. Data are presented as the mean ± SD. *P < 0.05, **P < 0.01, ***P < 0.001. *P‐*values were determined by two‐way ANOVA (D [right], G [right]).

To assess the impact of the deubiquitinating enzyme PSMD14 on LDHA protein stability, we measured LDHA protein levels and mRNA levels in PC cells following PSMD14 knockdown or overexpression using Western blotting and qRT‒PCR. The results revealed no significant changes in LDHA transcript levels in the knockdown PC cell lines or PSMD14‐overexpressing cells (Figure S7A, Supporting Information), but LDHA protein levels increased or decreased accordingly (**Figure** **3**A). Notably, although cell‐based experiments indicated that PSMD14 does not affect LDHA transcription, TCGA database analysis revealed a significant positive correlation between their mRNA levels in pancreatic cancer tissues (Figure S7B, Supporting Information). This discrepancy in expression between tissue and cellular levels may be associated with the complex tumor microenvironment characteristic of pancreatic cancer. Future studies could integrate single‐cell sequencing to further elucidate the specific regulatory networks within distinct cellular subpopulations of this microenvironment.

Protein stability studies revealed that LDHA protein expression increased in a time‐dependent manner after the cells were treated with the proteasome inhibitor MG132 (Figure 3B). Further experiments demonstrated that LDHA protein expression gradually increased with increasing PSMD14 expression (Figure 3C). In PC cells treated with the half‐life of the protein synthesis inhibitor CHX, the LDHA protein level was prolonged when MG132 was coadministered (Figure 3D).

To further investigate the degradation pathway of LDHA, we used the proteasome inhibitor MG132 to inhibit the ubiquitin–proteasome degradation pathway. Interestingly, in MG132‐treated PC cells, changes in PSMD14 expression did not significantly alter LDHA levels. Additionally, MG132 abrogated the decrease in LDHA protein levels caused by PSMD14 knockdown, whereas chloroquine (an autophagy–lysosome inhibitor) had no effect on LDHA levels under the same conditions (Figure 3E,F). These results indicate that PSMD14 regulates LDHA via the ubiquitin–proteasome pathway rather than the autophagy‒lysosome pathway. In CHX‐treated PC cell lines, PSMD14 overexpression prolonged the half‐life of exogenous LDHA protein (Figure 3G), whereas PSMD14 knockdown shortened the half‐life of exogenous LDHA protein (Figure 3H). The notion that PSMD14 mediates LDHA degradation is collectively supported by these discoveries.

### 2.5 PSMD14 Deubiquitinates LDHA

**Figure 4:**
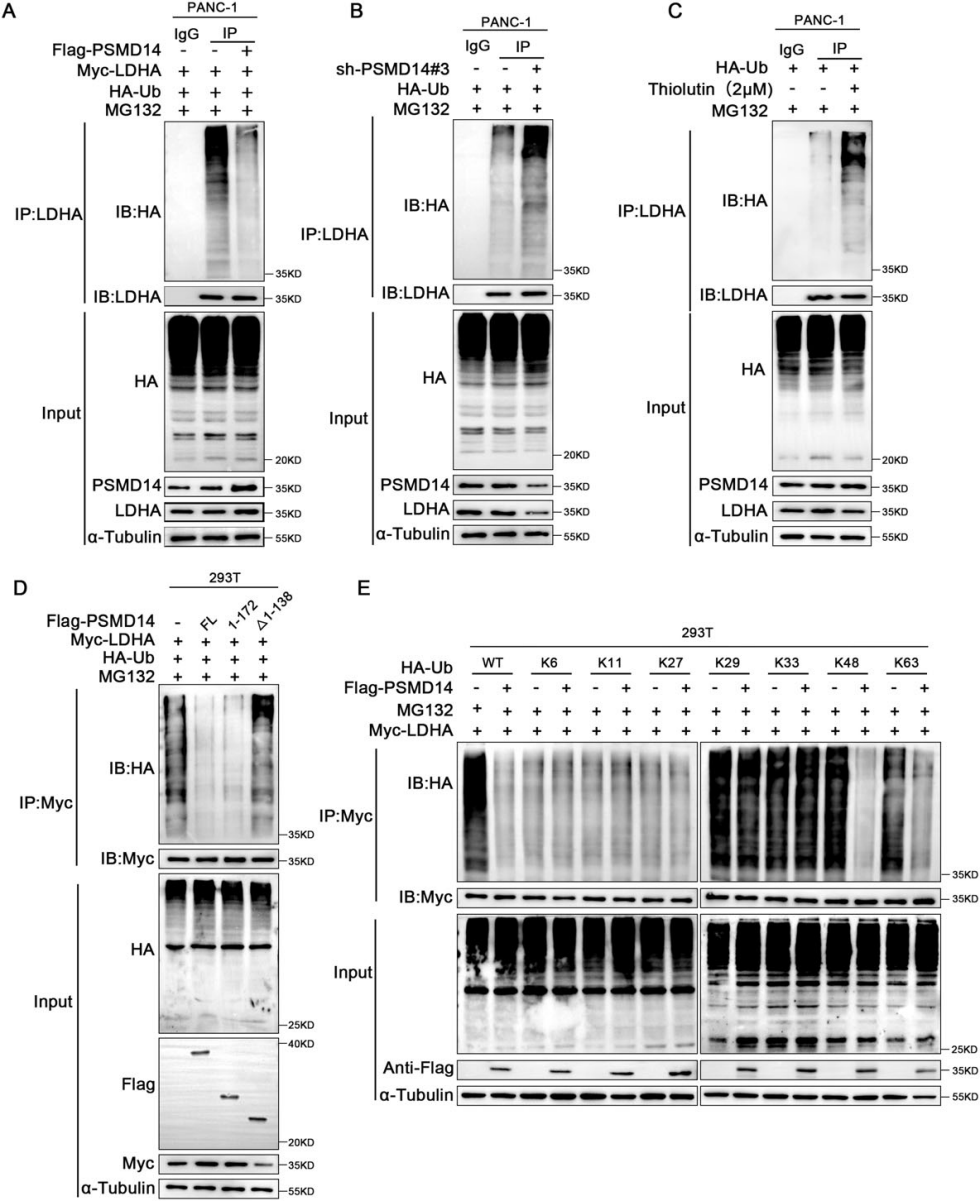
PSMD14 deubiquitinates LDHA. A,B) The Ub‐LDHA level was detected by immunoblotting with anti‐HA antibody in cells with PSMD14 overexpression or knockdown, following enrichment of the LDHA complex via immunoprecipitation; *n* = 3 biologically independent samples. C) PANC‐1 cells were treated with 2 µm thiolutin for 12 h, followed by immunoblotting with the indicated antibodies; *n* = 3 biologically independent samples. D) HEK‐293 T cells were transfected with plasmids encoding full‐length PSMD14 or its deletion mutants, and then immunoblotting was performed with the specified antibodies; *n* = 3 biologically independent samples. E) HEK 293 T cells were co‐transfected with HA‐WT, K6, K11, K27, K29, K33, K48, or K63 Ub, together with Flag‐PSMD14 and Myc‐LDHA. Subsequently, the cells were treated with MG132 (20 µm) for 8 h, and ubiquitination analysis was conducted on the cell lysates; *n* = 3 biologically independent samples.

As PSMD14 is a deubiquitinating enzyme, we investigated its potential to deubiquitinate LDHA. Ubiquitination assays revealed that LDHA ubiquitination levels were decreased in PSMD14‐overexpressing PC cells and increased in PSMD14‐knockdown PC cells (**Figure** **4**A,B). Given previous studies emphasizing the detailed inhibitory effect of thiolutin on PSMD14, we further evaluated its impact on LDHA deubiquitination. The Western blot results confirmed that the pharmacological inhibition of PSMD14 increased LDHA ubiquitination levels (Figure 4C). Additionally, domain‐based ubiquitination assays showed that both the MPN domain and the UBD of PSMD14 are needed for LDHA deubiquitination (Figure 4D). Transfection of HEK‐293T cells with HA‐Ub plasmids carrying different ubiquitin chains revealed that PSMD14 effectively removed K48‐ and K63‐linked ubiquitin chains from LDHA, revealing the mechanism by which PSMD14 stabilizes the LDHA protein (Figure 4E). In summary, PSMD14 was identified as a specific deubiquitinating enzyme that deubiquitinates and stabilizes LDHA.

### 2.6 PSMD14 Promotes Lipid Deposition in PC Cells via LDHA‐Driven Lactate Accumulation and Histone Lactylation

**Figure 5:**
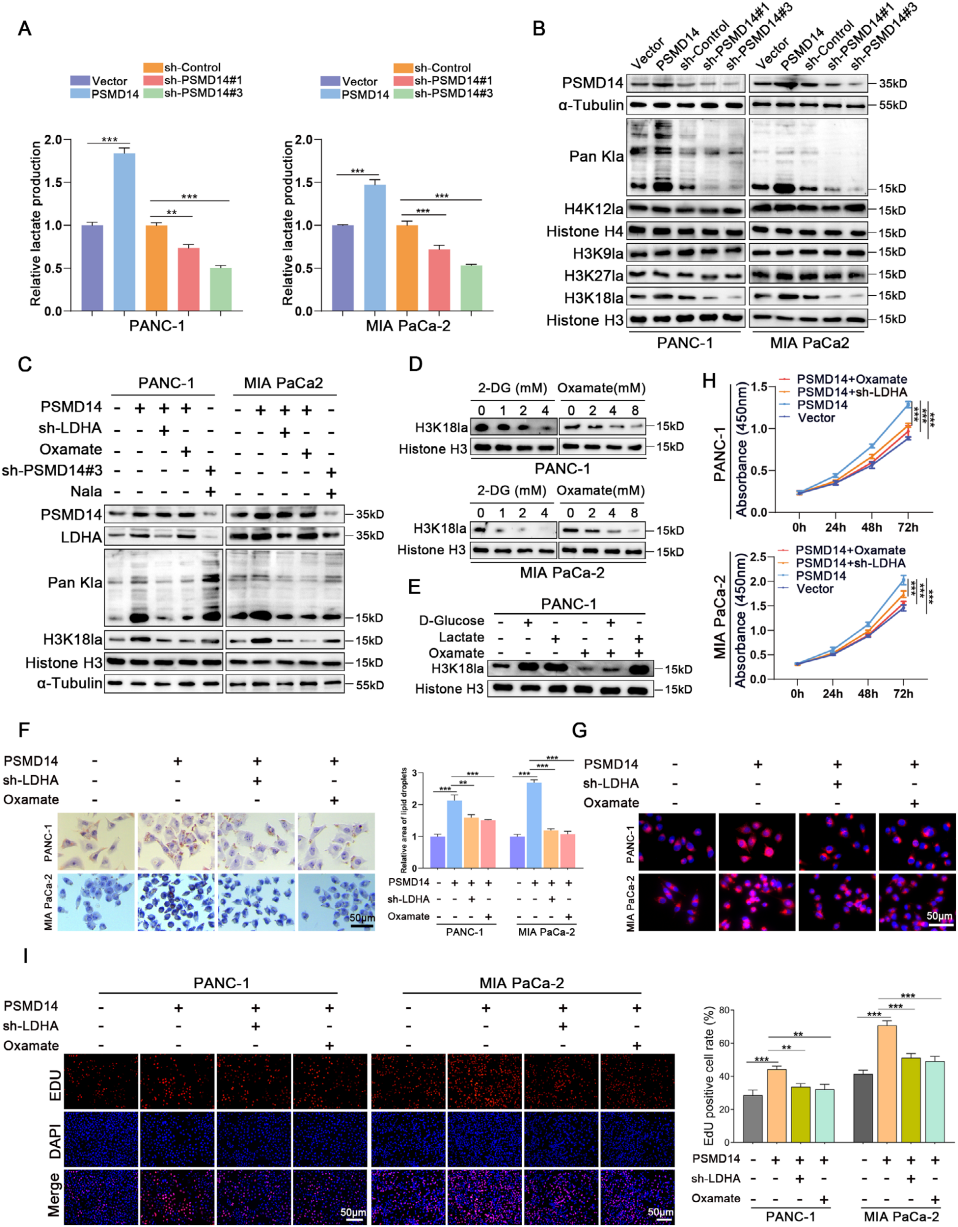
PSMD14 is critical for lipid deposition in pancreatic cancer cells through histone lactylation driven by LDHA‐mediated lactate accumulation. A) Detection of lactate levels in PANC‐1 and MIA PaCa‐2 cells with different PSMD14 contents; *n* = 3 biologically independent samples. B) Western blot analysis of Pan Kla, H4K12la, H3K9la, H3K18la, and H3K27la levels in PANC‐1 and MIA PaCa‐2 cells after PSMD14 upregulation or depletion; *n* = 3 biologically independent samples. C) In PC cells transfected with the oePSMD14 plasmid or sh‐LDHA, after treatment with Oxamate (8 mm) or Nala (20 mm) for 24 h, the lactylation level was analyzed by Western blot; *n* = 3 biologically independent samples. D) Western blot analysis of H3K18la expression in PANC‐1 and MIA PaCa‐2 cells cultured with different concentrations of 2‐DG or Oxamate for 24 h; *n* = 3 biologically independent samples. E) Western blot analysis of H3K18la protein levels in PANC‐1 cells induced by D‐Glucose (25 mm) or Lactate (10 mm) after the addition of glycolysis inhibitor Oxamate (8 mm); *n *= 3 biologically independent samples. F,G) In cells overexpressing PSMD14, after treatment with glycolysis inhibitor Oxamate (8 mm) for 24 h or LDHA knockdown, cell lipid accumulation was detected by Nile red and ORO staining; *n* = 3 biologically independent samples. H) Proliferation of PANC‐1 and MIA PaCa‐2 cells with PSMD14 overexpression followed by treatment with glycolysis inhibitor Oxamate (8 mm) for 24 h or LDHA knockdown was analyzed using CCK‐8 assay; *n* = 3 biologically independent samples. I) The proliferative capacity of PANC‐1 and MIA PaCa‐2 cells treated with glycolysis inhibitor Oxamate (8 mm) was detected by EdU staining; *n* = 3 biologically independent samples. Data are presented as the mean ± SD. *P < 0.05, **P < 0.01, ***P < 0.001. *P*‐values were determined by unpaired two‐tailed Student's *t‐*test (A), one‐way ANOVA (A,F,I), or two‐way ANOVA (H).

In the glycolytic pathway, LDHA plays an essential role by catalyzing the conversion of pyruvate into lactate.^[^ ^17^ ^]^ Lactate was historically considered a waste product of glycolysis. Recent studies, however, have shown that lactate is abundantly produced during the Warburg effect in the tumor microenvironment and can trigger histone lysine lactylation, which is a new epigenetic modification that stimulates gene copying directly from chromatin.^[^ ^18^ ^]^ To investigate whether PSMD14 influences lactate levels in PC cells and subsequently mediates histone lactylation, we first measured lactate levels in PSMD14‐overexpressing and PSMD14‐knockdown PC cells. The results proved that PSMD14 overexpression increased lactate levels, whereas PSMD14 knockdown reduced lactate levels (**Figure** **5**A). However, the increase in lactate levels induced by PSMD14 overexpression was significantly counteracted by the glycolytic inhibitor oxamate and LDHA knockdown (Figure S8A, Supporting Information). Collectively, these findings support the notion that PSMD14 increases lactate accumulation in PC cells through LDHA.

Elevated lactate levels can modify lysine residues on histones, leading to lactylation. Recently, histone lactylation has been reported to regulate various biological processes, including tumorigenesis, progression, immune evasion, and metabolic reprogramming in cancer cells.^[^ ^19^ ^]^ First, we examined total protein lactylation levels and several known histone lactylation sites in pancreatic cancer cells. Only H3K18la showed significant elevation following PSMD14 overexpression, leading us to focus on H3K18la's role in pancreatic cancer (Figure 5B). Subsequent experiments demonstrated that PSMD14‐mediated increases in H3K18la could be reversed by both LDHA knockdown and oxamate treatment, indicating lactate‐dependent regulation of this modification (Figure 5C). Notably, glycolytic inhibitors dose‐dependently decreased H3K18la levels in PC cells. Under glycolysis inhibition, exogenous glucose supplementation failed to increase histone lactylation levels, whereas treatment with lactate alone effectively restored these modifications (Figure 5D,E; Figure S8B, Supporting Information).

Considering the marked upregulation of lactate in pancreatic cancer (PC), it is crucial to investigate how lactate‐induced H3K18la modification regulates lipid biosynthesis in PC cells. Furthermore, targeted inhibition of LDHA and oxamate reduced the lipid accumulation induced by PSMD14 overexpression (Figure 5F,G) and repressed the proliferative capacity of cells (Figure 5H,I). In summary, our findings suggest that PSMD14‐mediated lipid deposition is associated with its modulation of histone lactylation. However, the precise molecular mechanisms underlying this relationship remain to be elucidated.

### 2.7 Histone Lactylation Activates ACLY Gene Transcription in PC

**Figure 6:**
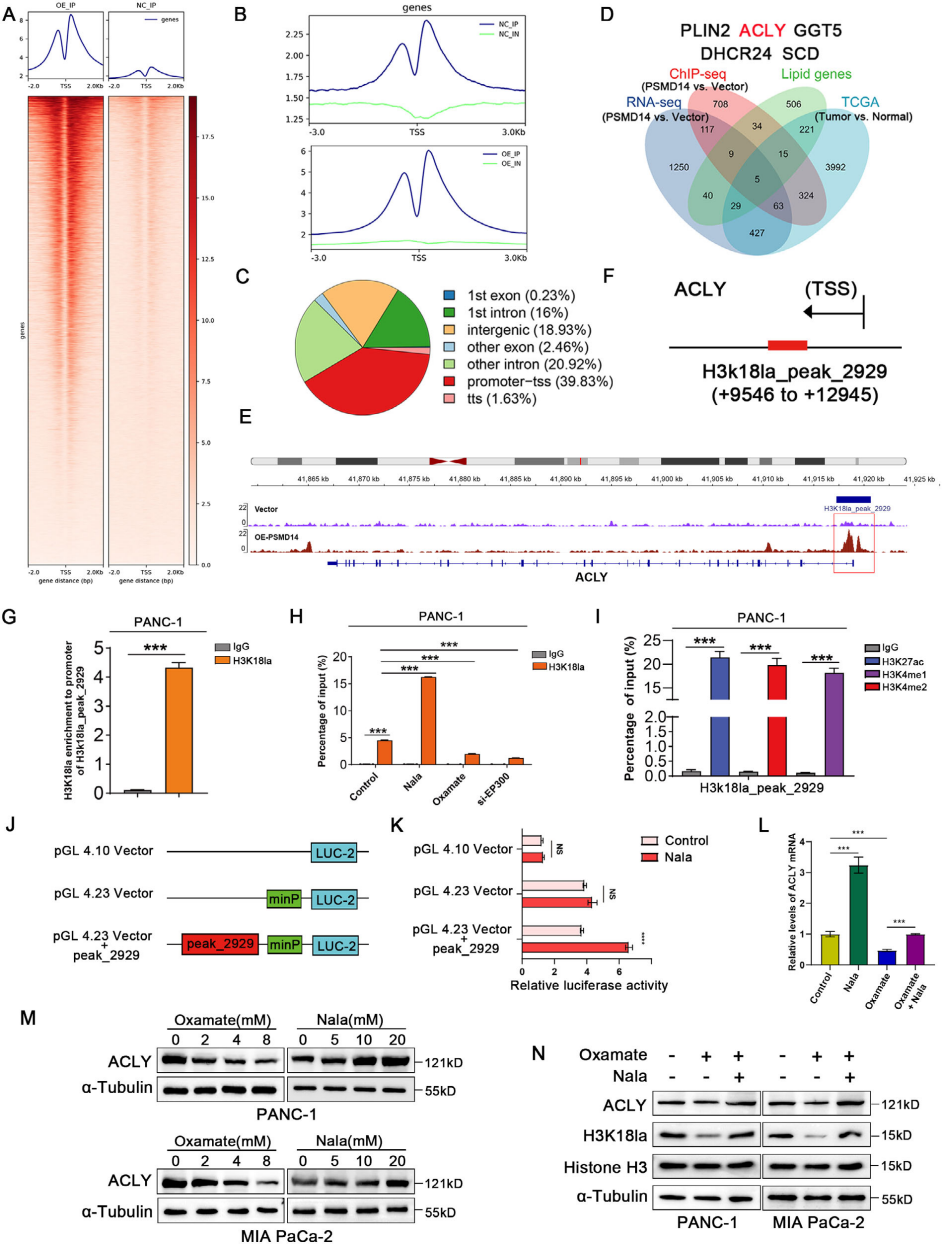
Histone lactylation activates ACLY transcription in pancreatic cancer. A,B) PANC‐1 cells were transfected with the oePSMD14 plasmid for ChIP‐seq assays to screen for H3K18la binding sites. The heatmap shows the distribution of H3K18la peaks near the translation start site (TSS). C) Genomic distribution of H3K18la. D) Venn diagram showing the combination of ChIP‐seq, RNA‐seq, lipid genes, and the TCGA database to identify potential downstream targets of H3K18la. E) Integrative Genomics Viewer (IGV) track diagram showing the enrichment peaks of H3K18la modification in ACLY ChIP‐seq data. F) Schematic diagram of potential enhancer binding sites in the ACLY sequence. G) DNA fragments from PANC‐1 cells were immunoprecipitated with H3K18la‐specific antibody and analyzed by qPCR using designated primers; *n* = 3 biologically independent samples. H) ChIP‐qPCR analysis of H3K18la status at ACLY gene peak_2929 in PANC‐1 cells treated with 20 mm Nala, 8 mm Oxamate, or transiently transfected with siRNA targeting EP300 for 24 h, with IgG and Input as controls; *n* = 3 biologically independent samples. I) ChIP‐PCR was used to detect the enrichment of enhancer markers H3K27ac, H3K4me1, and H3K4me2; *n* = 3 biologically independent samples. J) Schematic diagram of the dual‐luciferase reporter vector. K) Luciferase reporter assay in PANC‐1 cells transfected with different luciferase reporter vectors with or without 20 mm Nala; *n* = 3 biologically independent samples. L) qRT‐PCR was used to detect the expression level of ACLY in PANC‐1 cells after designated treatments; *n* = 3 biologically independent samples. M) Western blot analysis of ACLY expression in pancreatic cancer (PC) cells cultured with different concentrations of Oxamate or Nala; *n* = 3 biologically independent samples. N) Western blot analysis of ACLY expression and H3K18la level in PC cells treated with 8 mm Oxamate or 20 mm Nala; *n* = 3 biologically independent samples. Data are presented as the mean ± SD. *P < 0.05, **P < 0.01, ***P < 0.001. P values were determined by unpaired two‐tailed Student's *t‐*test (G,I,K) and one‐way ANOVA (H,L).

Histone lactylation is a new epigenetic modification that has been reported to regulate gene transcription directly from chromatin.^[^ ^18^, ^20^ ^]^ To further elucidate the regulatory function of H3K18la in gene expression, chromatin immunoprecipitation (ChIP‐seq) was performed using an anti‐H3K18la antibody in PC cells to discover gene targets that are regulated by H3K18la. The results revealed that H3K18la modifications were predominantly enriched near transcriptional start sites (TSS), regions critical for transcriptional regulation (**Figure** **6**A–C). By integrating ChIP‐seq, RNA‐seq, data from the TCGA database, and lipid metabolism‐related gene sets, we identified five differentially expressed genes (Figure 6D).

ACLY, a key gene that regulates lipid metabolism, may influence the malignant progression of PC by modulating metabolism and lipid synthesis. Visualization of the ChIP‐seq results using the integrative genomics viewer (IGV) genome browser revealed a single binding site (peak_2929) for H3K18la modification within the ACLY gene, located ≈+9546 to +12 945 bp downstream of the promoter (Figure 6E,F). ACLY expression showed significant positive correlations with lactate production‐related genes in the TCGA dataset (Figure S9A, Supporting Information). We further designed primers targeting this binding site and performed ChIP‒qPCR. The results revealed significant enrichment of H3K18la at peak_2929 compared with IgG (Figure 6G; Figure S9B, Supporting Information). In addition, application of Nala significantly increased the enrichment of H3K18la at peak_2929, while glycolysis inhibitors or EP300 siRNA reduced this enrichment. (Figure 6H; Figure S9C, Supporting Information). Thus, this site may be a critical locus for H3K18la modification of ACLY. Notably, peak_2929 is not located in the classical promoter region of ACLY (+99 to −2000 bp). Previous studies have suggested that H3K18la can regulate gene transcription at enhancer regions.^[^ ^21^ ^]^ We hypothesized that PSMD14‐mediated H3K18la might promote ACLY transcription by modulating its enhancer activity. First, we examined the enrichment of the enhancer markers H3K27ac, H3K4me1, and H3K4me2 at peak_2929 in PC cells. The results revealed significant enrichment of these markers at peak_2929 (Figure 6I; Figure S9D, Supporting Information), suggesting that this site is near an ACLY gene enhancer.

To elucidate the regulatory mechanism of H3K18la, we transfected pGL4.23 plasmids containing either the minp promoter alone or the minp promoter plus the H3K18la peak_2929 enhancer site into Nala‐induced cells and control cells. Dual‐luciferase reporter assays revealed that sodium lactate induction increased ACLY enhancer activity. However, this effect was not detected in the plasmids lacking the H3K18la peak_2929 site (Figure 6J,K; Figure S9E, Supporting Information). To precisely identify the main binding region of H3K18la, we constructed pGL4.23 reporter plasmids containing different binding fragments (Figure S9F, Supporting Information). Upon treatment with 20 mm Nala, a significant increase in luciferase activity was observed for pGL4.23_peak_2929_WT. Compared to other fragments, H3K18la exhibited stronger binding activity in the mutant constructs pGL4.23_peak_2929_mut #2 and pGL4.23_peak_2929_mut #5 (Figure S9G, Supporting Information).

Subsequently, we analyzed chromatin accessibility in PANC‐1 cells expressing wild‐type ACLY (WT) and mutants (#2 and #5, MUT) using ATAC‐seq. The results demonstrated a significant reduction in the distribution of sequencing reads around TSS in the ACLY MUT group (Figure S10A, Supporting Information). Further analysis revealed that the proportion of differentially accessible peaks located within promoter‐TSS regions was notably lower in the MUT group (33.56%) compared to the WT group (47.15%), and the overall density of open chromatin regions was markedly decreased (Figure S10B–E, Supporting Information). Among the 5372 identified differential peaks, 4366 exhibited reduced signals in the MUT group (Figure S10F,G, Supporting Information). Additionally, genome browser track analysis further confirmed a significant decrease in read counts proximal to promoter regions in the mutants (Figure S10H, Supporting Information). These results indicate that following ACLY mutations (#2 and #5), the enrichment of H3K18la in this region decreases, leading to reduced chromatin accessibility at the ACLY promoter region and consequently suppressing the transcriptional expression of the ACLY gene.

Notably, ACLY mRNA levels increased after Nala application, decreased upon glycolytic inhibitor treatment, and were partially restored by Nala supplementation (**Figure** **6**L). ACLY protein levels also increased in cells cultured with various concentrations of Nala. Conversely, ACLY protein levels decreased after glycolytic inhibitor treatment and were partially restored by Nala supplementation (Figure 6M,N). Overall, these data indicate that ACLY transcription is positively regulated by H3K18la.

### 2.8 Histone Lactylation Activates ACLY Gene Transcription and Mediates Lipid Deposition, Which is Critical for PC Cell Growth

Given the above findings, we next explored the role of ACLY, which is upregulated by H3K18la, in PC. Stable ACLY‐overexpressing cells were first established from the MIA PaCa‐2 and PANC‐1 cell lines (Figure S11A, Supporting Information). Increased ACLY expression promoted PC cell proliferation, and the glycolytic inhibitor oxamate partially abrogated the increased proliferative capacity induced by ACLY overexpression (Figure S11B,C, Supporting Information). Further investigations revealed that ACLY activation enhanced lipid deposition in PC cells, which could be reversed by oxamate treatment (Figure S11D,E, Supporting Information). To further validate the role of ACLY, we established a nude mouse xenograft tumor model. The results revealed that tumors in the ACLY‐overexpressing group were larger and heavier than those in the control group, but oxamate treatment reduced tumor size and weight in the ACLY‐overexpressing group (Figure S11F, Supporting Information).

### 2.9 Blocking PSMD14/LDHA/ACLY Pathway‐Mediated Lipid Synthesis Inhibits PC Progression

**Figure 7:**
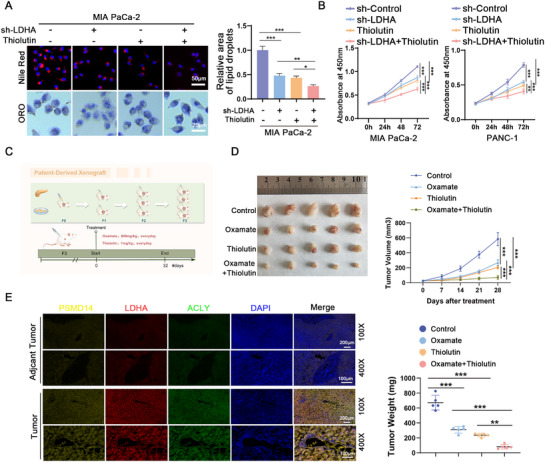
Blocking lipid synthesis mediated by the PSMD14/LDHA/ACLY pathway inhibits pancreatic cancer (PC) progression. A) MIA PaCa‐2 cells were transfected with shLDHA and then treated with or without Thiolutin (2 µm). Representative images of lipid accumulation measured by ORO staining and Nile red staining, along with corresponding quantitative analysis of staining; *n* = 3 biologically independent samples. B) Cell viability of PC cells after the indicated treatments was evaluated by CCK‐8 assay; *n* = 3 biologically independent samples. C) A patient‐derived xenograft (PDX) tumor model of pancreatic cancer was established, and starting from day 7, mice were intraperitoneally injected with 1 mg kg^−1^ Thiolutin or 500 mg kg^−1^ Oxamate daily. D) Images of tumor sizes in different groups, tumor growth curves of each group, and tumor weights; *n* = 5 mice per group. E) Multiplex fluorescence IHC was used to assess the protein expression levels of PSMD14, LDHA, and ACLY in PC and adjacent non‐cancerous tissues; *n* = 3 biologically independent samples. Data are presented as the mean ± SD. *P < 0.05, **P < 0.01, ***P < 0.001. *P*‐values were determined by one‐way ANOVA (A [right], D [bottom right]) or two‐way ANOVA (B,D [right]).

Next, we attempted to assess the therapeutic relevance of the PSMD14/LDHA/ACLY regulatory pathway in PC. Given previous studies highlighting the specific inhibitory effect of thiolutin on our finding that PSMD14‐mediated and PSMD14‐mediated lipid deposition is promoted by LDHA deubiquitination in PC cells,^[^ ^15a^ ^]^ we explored whether thiolutin could impede PC cell proliferation and PSMD14/LDHA/ACLY pathway‐mediated lipid deposition. First, we performed Oil Red O and Nile Red staining to examine the effects of thiolutin on lipid deposition and cell proliferation in PC cells. The results revealed that thiolutin treatment or LDHA knockdown significantly inhibited lipid accumulation in MIA PaCa‐2 cells, and this inhibitory effect was further increased through the combination of LDHA knockdown and thiolutin treatment (**Figure** **7**A). Concurrently, cell viability assays demonstrated that thiolutin treatment or LDHA depletion significantly reduced the proliferation of PANC‐1 and MIA PaCa‐2 cells, and the combination of both further inhibited cell proliferation (Figure 7B).

To validate the translational relevance of our in vitro findings, we established a pancreatic cancer patient‐derived xenograft (PDX) model system and evaluated the therapeutic efficacy of thiolutin against tumor progression (Figure 7C). In the PDX models, both thiolutin and oxamate monotherapy significantly inhibited xenograft tumor growth to similar degrees, whereas the combination of oxamate and thiolutin further inhibited tumor growth. Consistent with these results, changes in weight and tumor volume were also observed (Figure 7D). Multiplex fluorescence IHC staining revealed high‐intensity fluorescence signals for PSMD14, LDHA, and ACLY in PC tissues compared with adjacent normal tissues (Figure 7E).

In summary, our results provide new insights into the functional role of the PSMD14/LDHA/ACLY regulatory pathway in the progression and development of PC. An exploration of this pathway may aid in the development of novel therapeutic strategies for PC.

## 3 Discussion

PC, an extremely malignant cancer of the digestive system, has become an important public health challenge worldwide.^[^ ^1^, ^22^ ^]^ Among the hallmarks of tumor metabolic reprogramming, dysregulated lipid metabolism has been confirmed as a critical factor that promotes the malignant phenotype of tumors.^[^ ^6^, ^7^, ^23^ ^]^ Lipid synthesis, uptake, and storage are modified to continuously meet the energy demands of cancer cells and provide precursors for macromolecular biosynthesis.^[^ ^9^, ^24^ ^]^ The upregulation of key fatty acid synthesis enzymes such as ACLY, ACC, and FASN drives the abnormal activation of de novo fatty acid synthesis pathways.^[^ ^25^ ^]^ However, the core regulatory mechanisms underlying aberrant lipid metabolism in PC cells remain incompletely understood.

**Figure 8:**
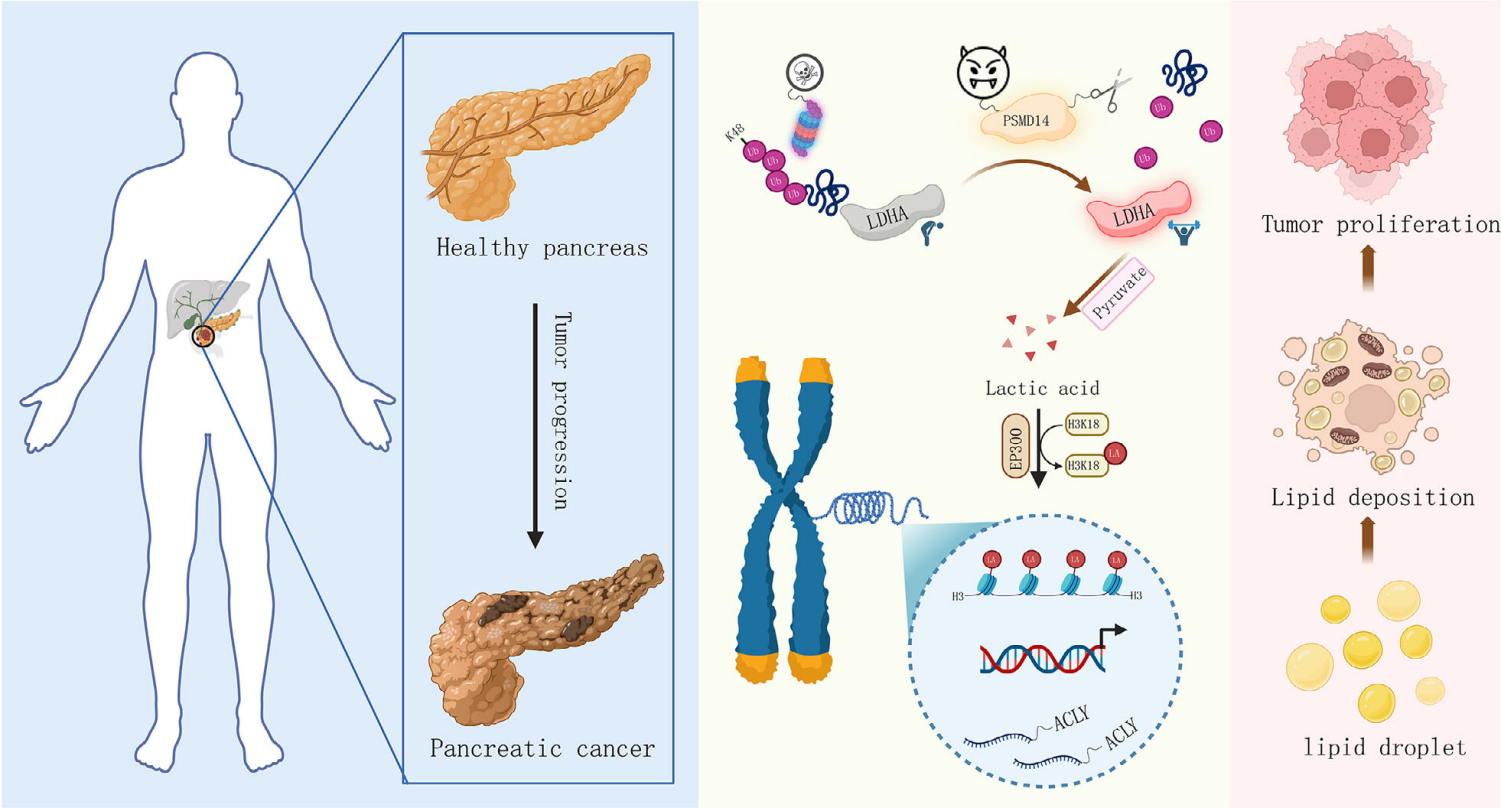
Schematic model demonstrating that the deubiquitinase PSMD14 increases lactate production by stabilizing LDHA, which leads to lactate‐induced histone hyperlactylation, increased ACLY transcription, and enhanced fatty acid metabolism, ultimately driving PC progression.

From an evolutionary perspective of cellular metabolic regulation, eukaryotes maintain metabolic homeostasis through dynamic and adaptive quality control systems.^[^ ^26^ ^]^ Among these systems, the UPS, as the primary regulatory system for protein homeostasis in eukaryotic cells, participates in the regulation of lipid metabolism‐related enzyme stability through spatiotemporally specific protein degradation mechanisms.^[^ ^27^ ^]^ Recent studies have shown that deubiquitination regulates key metabolic enzyme stability and lipid metabolism, such as the stabilization of the lipid droplet‐coating protein Perilipin 2, which modulates lipid storage in skeletal muscle.^[^ ^11^, ^28^ ^]^ ROS promotes colorectal tumorigenesis by keyly regulating lipid synthesis through a P53‐dependent mechanism involving the USP22‐FASN axis. Additionally, USP22 is a key regulator of de novo fatty acid synthesis, modulating the PPARγ‐ACLY/ACC axis to enhance the expression of ACC and ACLYs.^[^ ^29^ ^]^ The interaction between USP7 and RNF2, as well as their regulation of the PI3K/AKT signaling pathway, plays a role in modulating lipid metabolism and inflammation in alcoholic liver disease.^[^ ^30^ ^]^ On the basis of this theoretical framework, we hypothesize that the deubiquitinase network may play a central regulatory role in lipid metabolic reprogramming in PC by modulating the stability of lipid metabolic enzymes or the dynamics of lipid metabolism (**Figure** **8**). In our study, we found that PSMD14 upregulation promotes de novo fatty acid synthesis and cell proliferation in pancreatic cancer cells, leading to poor patient survival outcomes. Specifically, PSMD14 stabilizes downstream LDHA, thereby promoting lactate accumulation. Lactate accumulation increases ACLY activity through H3K18la modification, further driving lipid synthesis and tumor cell growth in PC.

With respect to the molecular mechanisms of PSMD14 in PC, we focused on exploring its role as a key deubiquitinase in the UPS and its regulatory mechanisms in tumor cell metabolism. Previous studies have focused primarily on its DNA damage repair functions and classical deubiquitination regulatory mechanisms, with limited research in PC confined to brief mentions of tumorigenesis and progression.^[^ ^15^, ^16^, ^31^ ^]^ In this study, the oncogenic function of PSMD14 in PC was revealed. Moreover, the ability of PSMD14 to regulate lipid metabolism in tumor cells was revealed for the first time. Previous research has shown that PSMD14 participates in the deubiquitination of the glycolytic rate‐limiting enzyme PKM2, promoting PKM2 nuclear translocation and glycolysis.^[^ ^32^ ^]^ Consistent with these findings, our findings demonstrate that PSMD14 directly stabilizes the LDHA protein through deubiquitination, significantly promoting lactate production and increasing glycolytic flux. LDHA catalyzes the conversion of pyruvate to lactate, representing a key step in the abnormal glycolytic metabolism of cancer cells.^[^ ^17^ ^]^ Related studies have shown that the activation of lactic acid promotes tumor progression mainly through two pathways: On one hand, lactate accumulation acidifies the microenvironment, facilitating immune evasion, drug resistance, tumor growth, and metastasis;^[^ ^33^ ^]^ on the other hand, lactate functions as a signaling molecule in epigenetic remodeling and serves as a substrate for histone lactylation.^[^ ^20^ ^]^

In recent years, significant attention has been given to lactylation, which is a key link between epigenetics and metabolism.^[^ ^19a^ ^]^ Until it was discovered that lactate can serve as a major carbon source for the tricarboxylic acid (TCA) cycle, lactate was considered a waste product of glycolysis.^[^ ^34^ ^]^ Professor Zhao Yingming's team discovered that lactate can regulate histone function through lactylation, further expanding the functional repertoire of lactate.^[^ ^35^ ^]^ Here, our study revealed that lactate accumulation driven by the PSMD14‐LDHA axis promotes H3K18 lactylation and revealed that histone lactylation drives lipid metabolic reprogramming in PC. Interestingly, whereas previous studies on histone lactylation modifications primarily focused on gene promoter regions,^[^ ^36^ ^]^ our results demonstrate that lactate‐driven H3K18la modification predominantly enriches enhancer regions of ACLY. This modification facilitates chromatin relaxation at the ACLY promoter area, thereby promoting gene transcription. Notably, ACLY, as a central hub connecting glucose metabolism and lipid synthesis, not only provides acetyl‐CoA substrates for lipid synthesis but also promotes histone acetylation through acetyl‐CoA generation, forming a “metabolic–epigenetic positive feedback loop”.^[^ ^37^ ^]^ This cascade amplification effect may be a key driver of rapid PC progression and chemotherapy resistance. Additionally, lipids play an essential role in tumors, not only by acts as sources for biosynthetic precursors and energy but also by participating in signal transduction, membrane structure formation, and tumor microenvironment regulation.^[^ ^7^, ^8^ ^]^ Our study demonstrated that transcriptional activation of ACLY directly enhances lipid synthesis in pancreatic cancer cells, a process closely linked to the malignant phenotype of pancreatic cancer and potentially explaining the poor prognosis observed in patients with high PSMD14 expression.

The UPS is a central pathway that regulates intracellular protein degradation and has become a significant target for cancer treatment.^[^ ^38^ ^]^ Currently, proteasome inhibitors such as bortezomib have achieved significant efficacy in the treatment of multiple myeloma.^[^ ^39^ ^]^ For the past few years, the natural product thiolutin has garnered attention because of its anti‐inflammatory, antiangiogenic, and antitumor activities.^[^ ^40^ ^]^ Notably, significant tumor inhibition was observed in a mouse model in which the PSMD14 inhibitor thiolutin was used or PSMD14 was knocked down. This research suggests that inhibiting PSMD14 may ameliorate PC by correcting tumor metabolism. Our study indicates that inhibiting PSMD14 may improve the therapeutic efficacy in pancreatic cancer, demonstrating potential for clinical application.

This study has certain limitations. Although existing research suggests that lipid deposition in the tumor microenvironment plays a significant role in immune resistance and immune cell function,^[^ ^41^ ^]^ the specific role of PSMD14 in this process remains to be explored. In particular, abnormal lipid metabolism in tumor cells may affect their interactions with immune cells (e.g., T cells and macrophages), reshape the tumor microenvironment, and promote immune evasion, a likely mechanism that requires further investigation. Research will concentrate on the molecular mechanisms by which lipid deposition regulates immune cell roles and cultivate combination immunotherapy strategies that target lipid metabolism.

In conclusion, an interaction network among deubiquitination, histone lactylation, and lipid metabolism regulatory genes was revealed in this study. This study provides new insights into the pathogenesis of PC and enhances our understanding of the link between energy metabolism and epigenetic regulation. These findings confirm a novel role of PSMD14 in cellular lipid metabolism, suggesting that the use of PSMD14 inhibitors may become a potential strategy for PC treatment and offering new research directions for clinical therapy.

## 4 Experimental Section

### Gene Expression Analysis Based on Tumor Database

Analyzing PSMD14 mRNA expression levels in pancreatic cancer based on the GEPIA database (http://gepia2.cancer‐pku.cn/). The experimental procedure was as follows: Selected the “Expression Analysis” module and used the “Expression DIY” feature. In the “Dataset” section, TCGA‐PAAD tumor samples were chosen, and GTEx Pancreas normal samples as controls. Entered “PSMD14” in the “Gene” input box. Parameters: |Log2FC| cutoff = 1, *p*‐value cutoff = 0.05 were set, and checked the “Log Scale” option (which applied log2(TPM + 1) transformation). Adjusted “Jitter Size” to 0.4. After submission, a differential expression boxplot and statistical results would be generated. Subsequently, on the Survival Analysis interface, the median expression level was used as the grouping cutoff (Group Cutoff = Median, with 50% in the high‐expression group and 50% in the low‐expression group) to evaluate the association between PSMD14 expression levels and overall survival (OS) as well as disease‐free survival in pancreatic cancer patients.

### Weighted Gene Co‐Expression Network Analysis (WGCNA) Analysis

WGCNA was employed to construct a weighted co‐expression network utilizing the GSE16515, GSE15471, and GSE28735 datasets obtained from the Gene Expression Omnibus (ncbi.nlm.nih.gov/gds). First, these three datasets were systematically integrated: data compatibility was achieved using the “inSilicoMerging” package in R software (version 4.2.1), and batch effects were corrected through the ComBat algorithm to generate a unified expression matrix. Subsequently, a scale‐free co‐expression network was constructed using a soft‐thresholding power of *β *= 8, and hierarchical clustering was performed using the dynamic tree cut algorithm (minimum module size set to 30). This process resulted in the identification of 21 stable modules (sensitivity set to 3 and module merging threshold at 0.25). During module‐trait relationship analysis, multiple testing correction was applied: for modules associated with tumor tissue characteristics, the Bonferroni correction was used to set the significance threshold (*P *< 0.05/21 = 0.002381). Modules with a high correlation were further refined by selecting genes with a module membership (MM) absolute value > 0.8. From the darkorange2 module, 239 core genes were identified. Finally, cross‐validation was performed with the deubiquitinating enzyme gene set (*n* = 282) retrieved from the iUUCD database (iuucd.biocuckoo.org), resulting in the prioritization of two candidate targets for further study: PSMD14 and PSMD7.

### GSEA Analysis

GSEA analysis was conducted based on RNA‐seq data from PSMD14 overexpression and control groups in PANC‐1 pancreatic cancer cells. Differentially expressed genes were identified using a threshold of *p* < 0.05, and their log2 fold change (log2FC) values were extracted. The analysis was performed using the clusterProfiler package (version 4.4.4) in R (version 4.2.1). Prior to the analysis, gene identifiers were standardized and converted (e.g., Ensembl IDs to gene symbols) using the org.Hs.eg.db annotation package to ensure compatibility with the reference gene sets. The reference gene set was obtained from the MSigDB database (c2.cp.kegg.v2022.1.Hs.symbols.gmt), accessed via the msigdbr package. The enrichment analysis employed a pre‐ranked gene set enrichment approach: all genes were ordered in descending order based on their log2FC values, and the default weighted enrichment statistic was applied. Significance was assessed through 10 000 permutations. Multiple testing correction (such as Bonferroni correction) was applied to the results. Pathways with significant enrichment were identified accordingly.

### Human Pancreatic Cancer Specimens

PC tissue samples (*n* = 60) were obtained from patients diagnosed between April 2020 and February 2023 at Guizhou Medical University Hospital and its affiliated Cancer Hospital (Guiyang, China). All specimens were confirmed as pancreatic ductal adenocarcinoma by pathological examination. The samples were collected from patients with complete clinicopathological information who had no concurrent malignancies. Throughout the study, patient privacy was strictly maintained. This research was approved by the Ethics Committee of Guizhou Medical University's Affiliated Hospital (Guiyang, China; approval number: Ethics Review No. 073 of 2019). Prior to sample collection, patients were thoroughly informed about the study requirements and provided written informed consent for the use of their clinical samples and associated data. To ensure consistency and comparability across subsequent analyses, all experiments—namely PCR, western blot (WB), and immunohistochemistry (IHC)—used samples derived from the same patient cohort. PCR and WB analyses were performed on tissues from these 60 patients, and IHC was conducted on corresponding tissue sections from the same specimens. All experiments were independently repeated three times to validate the reliability of the results.

### Cell Lines and Cell Culture

The human PC cell lines used in this study included AsPC‐1, BxPC‐3, MIA PaCa‐2, SW 1990, and PANC‐1 cells, and the human normal pancreatic epithelial cell line HPDEC6‐7, which was derived from cells cultured at the National Cancer Organization (Bethesda, USA). Human embryonic kidney 293T cells were obtained from the Cell Bank of the Chinese Academy of Sciences. In accordance with the nutritional requirements and growth features of the different cell lines, BxPC‐3C and AsPC‐1 cells were cultured in RPMI‐1640 medium (Gibco, USA) supplemented with 10% fetal bovine serum (FBS; Gibco, USA). MIA PaCa‐2, SW 1990, PANC‐1, HPDEC6‐7, and human 293T cells were cultured in medium supplemented with 10% FBS (Gibco, USA) in DMEM (Gibco, USA). All the cell lines survived under suitable conditions (37 °C, 5% CO2).

### Cell Infection and Transfection

BioWorks Bio (Shanghai, China) was commissioned to synthesize small interfering RNAs (siRNAs) targeting PSMD14 and plasmids used for the ubiquitination experiments, including HA‐Ub, HA‐K6, HA‐K11, HA‐K27, HA‐K29, HA‐K33, HA‐K48, and HA‐K63. GigaChem (Shanghai, China) constructed lentiviruses with short hairpin RNAs (shRNAs) targeting PSMD14 and LDHA and a control lentivirus, and PSMD14‐, LDHA‐, and ACLY‐overexpressing lentiviruses and a negative control lentivirus. All cells to be transfected were cultivated under appropriate culture conditions, and when the cells were grown to the appropriate density, the less harmful transfection reagent Lipofectamine 3000 (Invitrogen, L3000015) was used for plasmid and siRNA transfection. The reagents that were required for lentiviral infection were available from the manufacturer. The sequences of the siRNAs and shRNAs used are shown in Table S3 (Supporting Information).

### RNA Extraction and qRT‒PCR Analysis

Cell lines and PC tissues were obtained following standard procedures. RNA was first obtained from cell lines and PC tissues, and the RNA concentrations were determined for individual samples using an RNA kit (Omega, R6934‐01). The acquired RNA was mixed and reverse transcribed into cDNA using PrimeScript RT Master Mix (Takara, RR036A), and the expression levels of the target genes and α‐Tubulin were measured. Relative differences in gene expression were determined via the 2^‐ΔΔCt^ method. The primers used are listed in Table S4 (Supporting Information).

### RNA‐Seq

This experiment was conducted by Kangce Biotechnology (Wuhan, China). Initially, total RNA was extracted from each experimental group using TRIzol reagent. The quality and integrity of the RNA were assessed using an Agilent 2100 Bioanalyzer, ensuring that all samples had RNA Integrity Numbers (RIN) greater than 7. Subsequently, poly(A) tails of mRNA molecules were selectively purified using oligo dT magnetic beads. The purified mRNA was then fragmented using a fragmentation reagent, followed by reverse transcription to synthesize double‐stranded cDNA (ds cDNA) using random hexamer primers and reverse transcriptase. Adapters were ligated to the cDNA fragments of each group, and the libraries were amplified by PCR to enrich the fragments. After quality control and validation, the libraries were subjected to high‐throughput sequencing on an Illumina platform. Finally, differential gene expression analysis was performed using tools such as DESeq2 to compare the various sample groups, allowing the identification of significantly differentially expressed genes.

### ATAC‐Seq

ATAC‐seq technology was employed to analyze the impact of ACLY mutation on chromatin accessibility in pancreatic cancer PANC‐1 cells. When the cells reach the logarithmic growth phase, they were gently washed with pre‐cooled PBS, followed by centrifugation at 300 g for 5 min at 4 °C to remove the supernatant, repeated twice. Next, the cells were resuspended in pre‐cooled lysis buffer containing 0.1% IGEPAL CA‐630 and lysed on ice for 10 min to release intact nuclei. After centrifugation, the nuclei were washed once with cold PBS. The purified nuclei were then gently mixed with transposition reaction buffer containing Tn5 transposase and incubated at 37 °C for 30 min to facilitate cleavage of open chromatin regions and insertion of sequencing adapters. Following the reaction, SDS was added to terminate the process, and the DNA was purified using magnetic beads. Subsequent PCR amplification was performed with Nextera primers for 5 to 7 cycles, and the amplified library was purified again with magnetic beads. Finally, Qubit fluorometry was used for concentration measurement, and Bioanalyzer analysis assessed fragment size distribution, ensuring high‐quality library preparation for downstream sequencing analysis.

### Western Blot Analysis

First, the cells or tissues were lysed in RIPA lysis solution (Solarbio, China) containing protease inhibitors (Solarbio, China) and centrifuged. The supernatant was collected, and the total protein concentrations were determined. Proteins were separated via SDS‒PAGE, followed by extraction of the desired proteins and transfer to a PVDF membrane (Millipore, USA). The PVDF membranes were blocked for 2 h at room temperature with skim milk and incubated overnight at 4 °C, after which they were incubated with the corresponding primary antibodies. The PVDF membranes were washed 3 times after primary antibody incubation and then incubated with the corresponding secondary antibody for 1 h at room temperature. Finally, the PVDF membrane was washed again and then detected using a chemiluminescence imaging system (Tanon 5200) and enhanced chemiluminescence solution. The primary antibodies used in this study are listed in Table S5 (Supporting Information).

### Immunohistochemistry (IHC)

Deparaffinization was essential for the immunohistochemical staining of paraffin‐embedded tissues, followed by hydration and antigen repair for antigenic epitope exposure, coincubation with the appropriate primary antibody, color development via DAB staining, and then visualization under a microscope and imaging. The staining intensity was scored as follows: 0 (negative staining), 1 (weak staining), 2 (moderate staining), and 3 (vigorous staining). The positive staining area was presented as a percentage of the total tissue area and was scored as 0 (0–5%), 1 (5–25%), 2 (26–50%), 3 (51–75%), and 4 (76–100%), respectively. The final IHC score was then calculated by multiplying the two scores. Final staining scores ≤ 6 were considered low expression, and scores > 6 were considered high expression. To ensure the accuracy of the results, two pathologists, who were unaware of all the patients’ clinical information, were involved in the analysis and scoring of the IHC staining results.

### Immunofluorescence (IF) Staining

PC cells were taken out of the incubator, fixed with 4% paraformaldehyde at room temperature for 15 min, and then washed with PBS. The cells were subsequently permeabilized with 0.3% Triton X‐100 (Servicebio, GC204003) for 10 min at room temperature and then washed thoroughly. After being blocked with 5% BSA for 1 h, the cells were incubated with the appropriate primary antibody overnight at 4 °C. Next, the cells were stained with a mixture of fluorescent secondary antibody and DAPI (Solarbio, C0065). The cells were subsequently observed and imaged under a confocal microscope (Japan, Nikon/AXR).

### Proximity Ligation Assay (PLA)

In pancreatic cancer cell lines PANC‐1 and MIA PaCa‐2, the NaveniFlex Cell MR Proximity Ligation System (Navinci Diagnostics, Sweden) was used to detect protein–protein interactions between PSMD14 and LDHA. All primary antibodies were rabbit or mouse polyclonal antibodies obtained from Proteintech, used at the recommended concentrations for immunocytochemistry. Cells were cultured on sterile glass slides, fixed with 4% paraformaldehyde for 15 min, and permeabilized with 0.1% Triton X‐100 at room temperature for 10 min. In a humidified chamber at 37 °C, cells were blocked with the provided blocking buffer for 1 h. Subsequently, the cells were incubated with a mixture of two primary antibodies against PSMD14 and LDHA at 37 °C for 1 h or overnight at 4 °C. After washing, the cells were incubated with 1:40 diluted NaveniFlex M1 and R2 proximity probes for 1 h, followed by washing with warm TBS‐T. The proximity ligation and rolling circle amplification reactions were performed according to the manufacturer's protocol. Nuclei were counterstained with DAPI. After washing with TBS, slides were mounted with an antifade mounting medium. Fluorescence imaging was performed using a confocal fluorescence microscope to visualize Cy3 (proximity signal) and DAPI signals.

### EdU Assay

Prior to the start of the experiment, PC cells were inoculated in 24‐well plates at 3 × 10^4^ cells well^−1^ and incubated with 10 µm EdU solution (Servicebio, G1602) for 2 h. The cells were fixed with 4% paraformaldehyde, and the cell membranes were then permeabilized with 0.3% Triton X‐100 (10 min). The nuclei of the cells were stained with DAPI (Solarbio, C0065), and click reaction mixtures were used to stain the cells.

### CCK‐8 Assay

PC cells were subjected to different treatments according to the experimental design. The cells were seeded in a 96‐well plate at a density of 3 × 10^3^ cells per well (with 5 replicate wells per group) and cultured for various durations: 0, 24, 48, and 72 h. Subsequently, 10 µL of CCK‐8 solution (Biosharp, BS350B) was added to each well at the defined time points, followed by incubation for an additional 2 h. Finally, the absorbance of each sample was measured at 450 nm using a microplate reader.

### Flow Cytometry for Cell Cycle Analysis

PC cells were seeded in 6‐well plates and treated accordingly. After digestion and centrifugation, the cells were fixed overnight at 4 °C with prechilled 70% ethanol. The cells were then stained with RNase A and propidium iodide (PI), incubated for 30 min, and analyzed using a flow cytometer (Agilent, China) according to the cell cycle protocol (Solarbio, CA1510).

### Lactate Quantification

The cultured cells were collected, and samples were processed following the manufacturer's protocol (Biosharp, BL868B). Following the addition of extraction buffer, the mixture was centrifuged, and the supernatant was collected for lactate level measurement via a microassay method.

### Transmission Electron Microscopy (TEM)

PC cells were washed with PBS and incubated in a 2.5% glutaraldehyde (Servicebio, G1102) solution in an incubator for ≈5 min. The cells were then gently detached using a cell scraper and centrifuged (at no more than 3000 rpm min^−1^) for ≈2 min. The cells were resuspended in 2.5% glutaraldehyde and observed via TEM.

### Oil Red O and Nile Red Staining Assays

Cultivated cells or tissue cryosections were fixed with 4% paraformaldehyde. A freshly prepared Oil Red O working solution was used to stain the samples for 15 min, and the cell nuclei were counterstained with hematoxylin. For Nile Red staining, cultured cells were washed and incubated with Nile Red solution for an appropriate duration. After thorough washing, nuclei were counterstained with DAPI, and cellular morphology was analyzed by fluorescence microscopy.

### Free Fatty Acid, Triglyceride (TG), and Cholesterol Assays

Measurements were performed according to the manufacturer's protocols. A free fatty acid assay kit (Beyotime, S0215S) was used to calculate free fatty acid levels, a TG assay kit (Beyotime, S0219S) was used for TG quantification, and a cholesterol assay kit (Beyotime, S0211S) was used for cholesterol quantification. Tumor tissues or cultured cells were lysed using an ultrasonic homogenizer, followed by high‐speed centrifugation at 4 °C. The supernatant was obtained, added to the wells of a 96‐well plate, and incubated in the dark. The absorbance at 570 nm was measured using a microplate reader (Thermo, USA), and the quantification was performed accordingly.

### Coimmunoprecipitation (Co‐IP)

Cell samples for Co‐IP were collected and lysed using a mild NP‐40 lysis buffer (Solarbio, N8032) supplemented with protease inhibitors. The total cell lysates were precleared with the corresponding IgG for 2 h, followed by incubation with the specific antibody overnight at 4 °C. The next day, protein A/G magnetized beads (Beyotime, P2108) were added to the mixture, which was subsequently incubated for an additional 2 h. The immunoprecipitated complexes were enriched using a magnetic stand, washed thoroughly, and subjected to immunoblotting following standard procedures.

### Protein Stability Assay

To investigate the half‐life of LDHA, PC cells were precultured and allowed to grow under optimal conditions. The cells were then treated with 100 µm narramycin A (Sigma, 66‐81‐9) for the specified durations. The protein samples were subsequently analyzed through Western blotting.

### In Vivo Deubiquitination Assay

PANC‐1 cells and HEK293T cells were transfected with HA‐Ub, Flag‐PSMD14, and Myc‐LDHA plasmids in 6‐well plates. After 48 hours, the cells were treated with 20 µm MG132 for 8 h, followed by cell lysis. Protein–antibody complexes were isolated using Co‐IP, and the ubiquitination levels of the target proteins were analyzed by using Western blotting.

### GST Pull‐Down Assay

The pGEX‐6P‐1 plasmid was used to construct GST fusion proteins, which were then transformed into *E. coli* BL21 cells for protein expression induction using isopropyl‐beta‐D‐1‐thiogalactopyranoside (IPTG). The GST fusion proteins were incubated with glutathione‒agarose beads (Beyotime, P2260S) to obtain purified GSH‒GST fusion protein complexes. Cell lysates containing the target proteins were prepared and mixed with the GST fusion protein‐bound beads, followed by incubation at 4 °C for 2 h. The beads were then pelleted, washed with GST binding buffer, and subjected to Western blot analysis with the appropriate antibodies.

### Chromatin Immunoprecipitation (ChIP) and ChIP‒qPCR

ChIP assays were performed according to the manufacturer's protocol using the CST (91820S) kit. PANC‐1 or MIA PaCa‐2 cells were cultured under optimal conditions. Cells were crosslinked with 1% formaldehyde (37%) for 10 min at room temp, then glycine was added to quench. Cells were collected, resuspended in lysis buffer on ice for 10 min, then lysed with nuclear lysis buffer. Chromatin was fragmented with micrococcal nuclease at 37 °C for 20 min, terminated with EDTA. Sonication was performed at 30% intensity, 20 s pulse^−1^, 30‐sec intervals, for 3 cycles to obtain ≈300 bp fragments, then centrifuged at 16 000 × g for 10 min. From the chromatin, 50 µL was incubated with water, NaCl, and RNase A at 37 °C for 30 min; then Proteinase K was added, and samples were incubated at 65 °C for 2 h for protein degradation. DNA was purified using spin columns and eluted; concentration was measured at 260 nm (50–200 µg mL^−1^). An input control was reserved with 10 µL. For IP, 10 µg chromatin was divided; 5 µg anti‐H3K18la (Jingjie, PTM‐1427 RM) or IgG (CST, 3900) was added, rotated overnight at 4 °C. The next day, magnetic beads were added and incubated for 2 h at 4 °C. Beads were washed via magnetic separation; 150 µL ChIP buffer was added. Input was incubated at room temperature; IP and control samples were decrosslinked at 65 °C for 30 min. Supernatant was collected, then 6 µL NaCl and 2 µL Proteinase K were added; incubated at 65 °C for 2 h for crosslink reversal. DNA was purified for downstream analysis. The entire process was repeated thrice for reproducibility. ChIP‐seq was conducted by Kangce Biotechnology (Wuhan, China). ChIP‐qPCR used TB Green Premix (Takara, CN830A) on a CFX96 (Bio‐Rad). Primer sequences are listed in Table S4 (Supporting Information).

### Dual‐Luciferase Reporter Gene Assay

PC cells were transfected with plasmids containing the constructed reporter gene vector and Renilla luciferase vector using the low‐cytotoxicity Lipofectamine 3000 reagent (L3000015, Invitrogen), then cultured in the presence or absence of Nala (20 mm). After the cell culture medium was removed, cell lysis buffer was added to completely lyse the cells, and the supernatant was collected. Subsequent detection was performed via the dual‐luciferase reporter assay kit (Promega, E1960). Each sample was mixed with 100 µL of firefly luciferase detection reagent, and the relative light units (RLUs) were measured using a multifunctional microplate reader. Subsequently, 100 µL of Renilla luciferase detection reagent was added to the same sample, and the RLU values were measured again. The activation of the target reporter gene in different samples was evaluated by comparing the ratio of firefly luciferase RLUs to Renilla luciferase RLUs.

### In Vivo Mouse Experiments

Female BALB/c and NOD‐SCID mice aged 6–8 weeks were purchased from GemPharmatech (Chengdu, China) and housed in a standard specific pathogen‐free (SPF) facility. The animal experiments were approved by the Animal Ethics Committee of Guizhou Medical University (Guiyang, China; Approval No. 19 000 672) and were performed following the National Institutes of Health (NIH) guidelines for the care and access of laboratory animals. The mice were randomly divided into groups, with 5 mice in each group. PANC‐1 cells (1 × 10⁶) subjected to stable ACLY‐overexpressing/control PANC‐1 cells or different treatments (1 × 10⁶) were resuspended in 100 µL of PBS and injected into the right flank of each mouse subcutaneously. After one week, drugs were administered intraperitoneally according to the experimental design. Mouse health status and palpable tumor volume were monitored weekly.

To establish the PDX model, fresh human PC tumor tissue pieces were subcutaneously transplanted into NOD‐SCID mice. Once an appropriate volume was reached, the tumors were excised, divided into equal pieces. These tumor pieces were then transplanted into a new generation of NOD‐SCID mice. After this process was repeated three times, a balanced PC PDX model was successfully created. When the tumor volume was reached, NOD‐SCID mice were divided into control, oxamate‐treated, thiolutin‐treated, and oxamate plus thiolutin‐treated groups. In accordance with the experimental protocol, the mice were intraperitoneally injected with thiolutin (MCE, 1 mg kg^−1^, once daily for 28 days) or oxamate (MCE, 500 mg mouse^−1^, once every day for 28 days). Mouse health status and palpable tumor volume were monitored weekly. The tumor volume (mm^3^) was estimated as follows: tumor volume = (length × width^2^)/2. When the tumor size reached the experimental endpoint or when ulceration occurred, the mice were anesthetized and euthanized, and the tumors were collected for further analysis.

### Statistical Analysis

Statistical analysis was performed with GraphPad Prism 10.1.2 software (GraphPad Software). The correlations between clinicopathological characteristics and PSMD14 expression were determined using the chi‐square test. For skewed data, the median (interquartile range) was used. Comparisons between two groups were performed via Student's *t*‐test (two‐tailed), and comparisons among multiple groups were performed via ANOVA. Survival analysis was conducted via the Kaplan–Meier method, and differences between groups were analyzed via the log‐rank test. Cox proportional hazards models were used for multivariate analysis to explore the relationship between survival time and PSMD14 expression in PC patients. All data were presented as the mean ± SD. A *p*‐value < 0.05 was considered statistically significant (*p < 0.05, **p < 0.01, ***p < 0.001). Non‐significant results (ns) were defined as *p* ≥ 0.05.

### Ethics Approval

The study was approved by the Human Ethics Committee of the Affiliated Hospital of Guizhou Medical University and the Animal Ethics Committee of Guizhou Medical University. All subjects were informed of the study's purpose and provided written informed consent.

## Conflict of Interest

The authors declare no conflict of interest.

## Author Contributions

R.‐S.L., L.‐K.R., and X.‐B.F. contributed equally to this work. Conception and design were done by Y.‐Z.P. X.W. Experimental research was done by R.‐S.L., L.‐K.R., X.‐B.F., and J.‐Y.H. Collection of clinical samples was done by C.W. Animal model construction was done by S.‐B.L. and C.‐H.Z. Data analysis was done by R.‐S.L., L.‐K.R., X.‐B.F., S.‐B.L., Y.‐J.G., C.‐H.Z., and P.L. The Manuscript was written by R.‐S.L. Research supervision was done by Y.‐Z.P., X.W., and C.‐H.Z. Y.‐Z.P. and X.W. dealt with funding support. All authors have read and approved the final manuscript.

## Supporting information



Supporting Information

Supporting Information

Supporting Information

Supporting Information

Supporting Information

Supporting Information

Supporting Information

## Data Availability

The data that support the findings of this study are available from the corresponding author upon reasonable request.
