# Supplementary material for: PSMD14‐Mediated LDHA Deubiquitination Upregulates ACLY Expression via H3K18 Lactylation to Promote Lipid Synthesis and Pancreatic Cancer Progression
Source: Adv Sci (Weinh). 2025 Oct 6;12(44):e05762. doi: 10.1002/advs.202505762 (PMC12667490; doi:10.1002/advs.202505762)
Supplement: Supplementary file 1 — Supporting Information [file ADVS-12-e05762-s006.docx]

**Supplementary Information**

**PSMD14-Mediated LDHA Deubiquitination Upregulates ACLY Expression via H3K18 Lactylation to Promote Lipid Synthesis and Pancreatic Cancer Progression**

1. **Supplementary Figures:**

**Supplementary Figure S1** Construction of weighted gene co-expression network (WGCNA) and identification of key modules.

**Supplementary Figure S2** LASSO analysis identifies key regulatory factors of lipid metabolism in pancreatic cancer.

**Supplementary Figure S3** Overexpression of PSMD14 in pancreatic cancer (PC) tissues and cell lines.

**Supplementary Figure S4** PSMD14 shows a positive correlation with the expression of key genes involved in fatty acid synthesis.

**Supplementary Figure S5** PSMD14 enhances the proliferation of pancreatic cancer cells both in vitro and in vivo.

**Supplementary Figure S6** Prediction and verification of the interaction between PSMD14 and LDHA.

**Supplementary Figure S7** Effect of PSMD14 on LDHA protein stability.

**Supplementary Figure S8** PSMD14 mediates lactate accumulation and histone lactylation in pancreatic cancer (PC).

**Supplementary Figure S9** Histone lactylation drives ACLY gene transcription activation in pancreatic cancer.

**Supplementary Figure S10** Effect of ACLY enhancer site mutation on chromatin accessibility.

**Supplementary Figure S11** ACLY transcription-mediated lipid deposition is critical for pancreatic cancer (PC) cells.

1. **Supplementary Tables (refer to the corresponding supplementary file):**

**Supplementary Table 1** Correlation Between PSMD14 Expression and Clinicopathological Characteristics of Pancreatic Cancer Patients.

**Supplementary Table 2** Cox Regression Analysis of Clinicopathological Characteristics and Prognosis in Pancreatic Cancer.

**Supplementary Table 3** siRNA sequences used in our experiement.

**Supplementary Table 4** Primers used in this study.

**Supplementary Table 4** Primary antibodies used in this study.

.
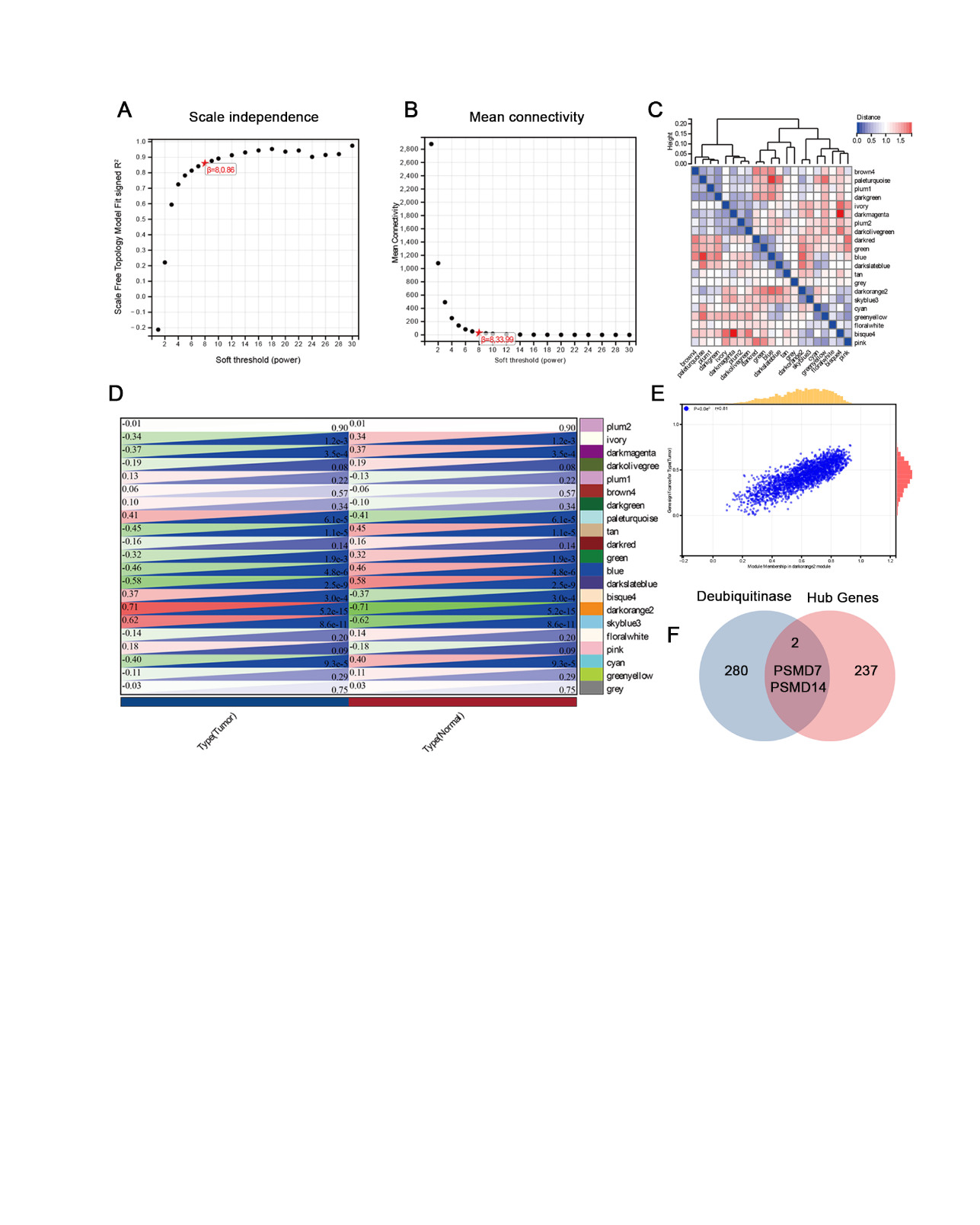


**Supplementary Figure S1 Construction of weighted gene co-expression network (WGCNA) and identification of key modules. (A, B)** Establishment of the optimal soft threshold. **(C)** Hierarchical clustering dendrogram of genes based on topological overlap matrix (TOM). **(D)** Correlation map between modules and phenotypic characteristics. **(E)** Identification of associations between genes, modules, and trait correlations. **(F)** Venn diagram illustrating the intersection of deubiquitinase dataset and core genes.


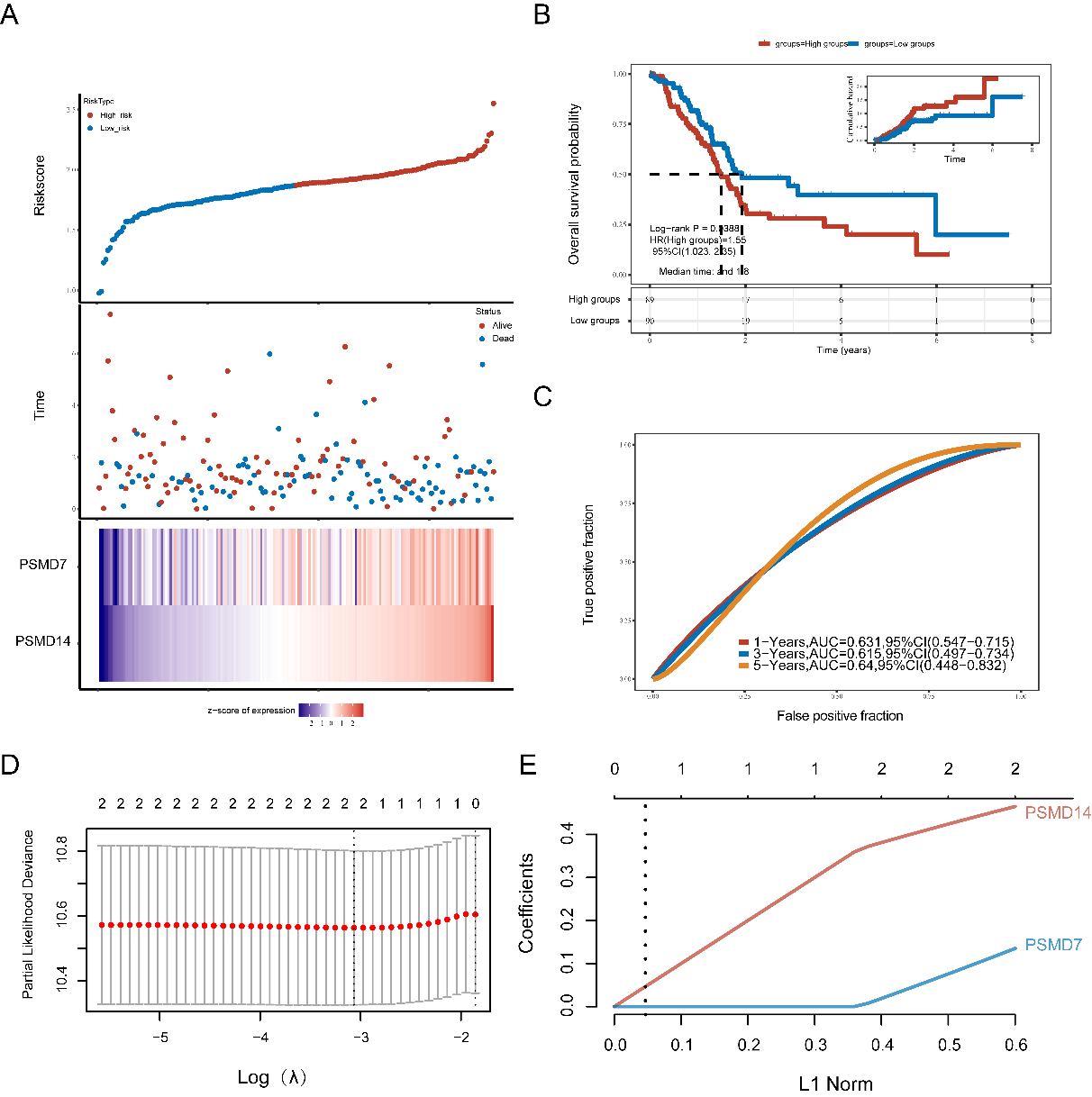


**Supplementary Figure S2 LASSO analysis identifies key regulatory factors of lipid metabolism in pancreatic cancer.​(A)** Association between gene expression and survival time, as well as survival status, in TCGA data. **(B)** Kaplan-Meier (KM) survival curve derived from TCGA data.​**(C)** ROC curves and corresponding AUC values at different time points. **(D)** Partial likelihood deviance plot of LASSO regression. **(E)** Coefficient path diagram of LASSO regression.

**
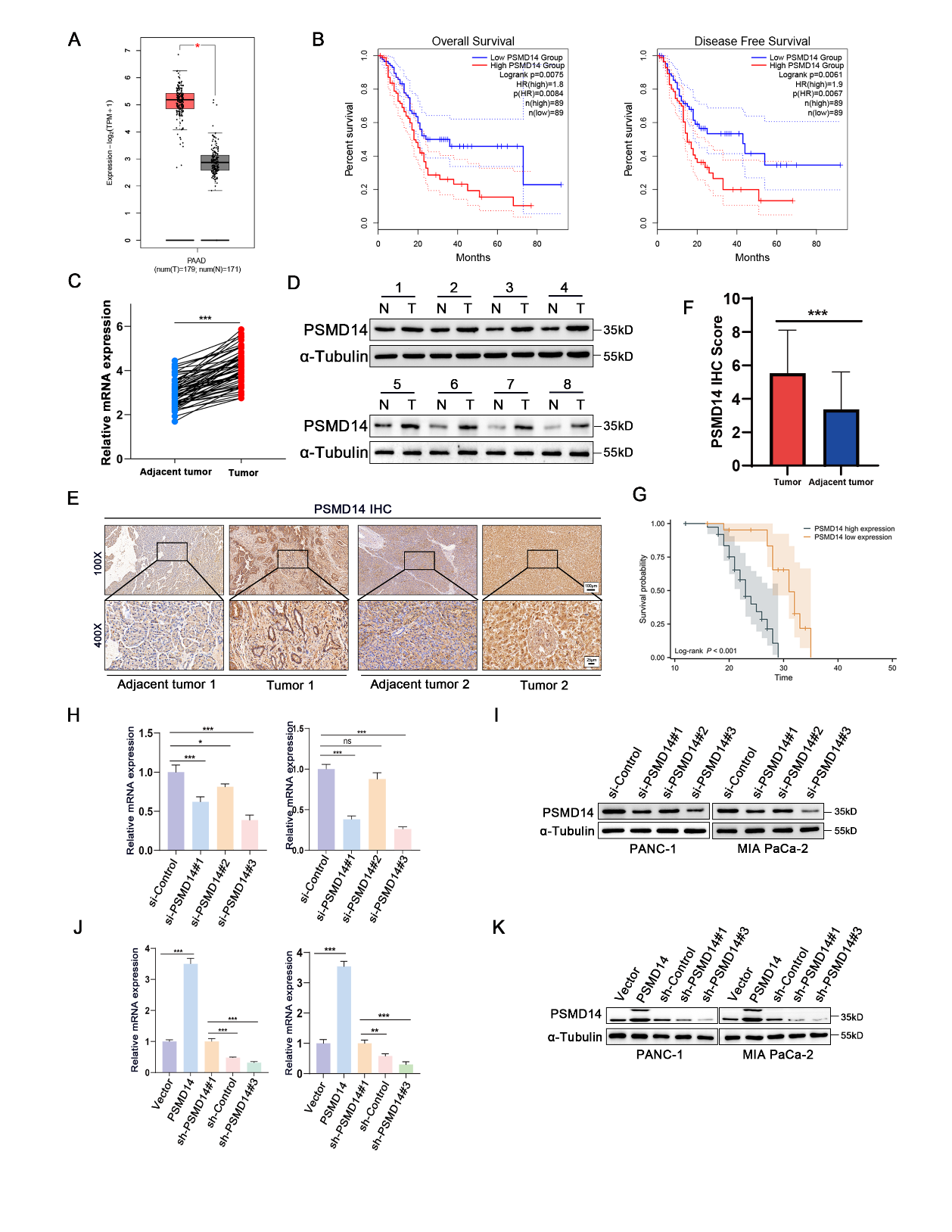
**

**Supplementary Figure S3 Overexpression of PSMD14 in pancreatic cancer (PC) tissues and cell lines. (A)** In the TCGA database, PSMD14 exhibits high expression in pancreatic cancer. **(B)** In the TCGA database, high PSMD14 expression is associated with poor prognosis in clinical PC patients.​**(C-E)** qRT-PCR (C), Western blot (D), and IHC (E) were performed to detect the expression level of PSMD14 in PC and adjacent non-tumor tissues; n = 60 biologically independent samples. **(F)** IHC scores of PSMD14 in PC and adjacent tissues.​**(G)** Kaplan-Meier analysis of overall survival in the PSMD14 low-expression and high-expression groups. **(H, I)** qRT-PCR and Western blot were used to assess the transfection efficiency of PSMD14 small interfering RNA; n = 3 biologically independent samples. **(J, K)** qRT-PCR and Western blot were employed to determine the transfection efficiency of PSMD14 overexpression or knockdown; n = 3 biologically independent samples.​Data are presented as mean±SD. *p < 0.05, **p < 0.01, ***p < 0.001. P values were calculated using paired two-tailed Student's t-test (C, F), one-way ANOVA (H, J), and Log-rank (Mantel-Cox) test (G).

**
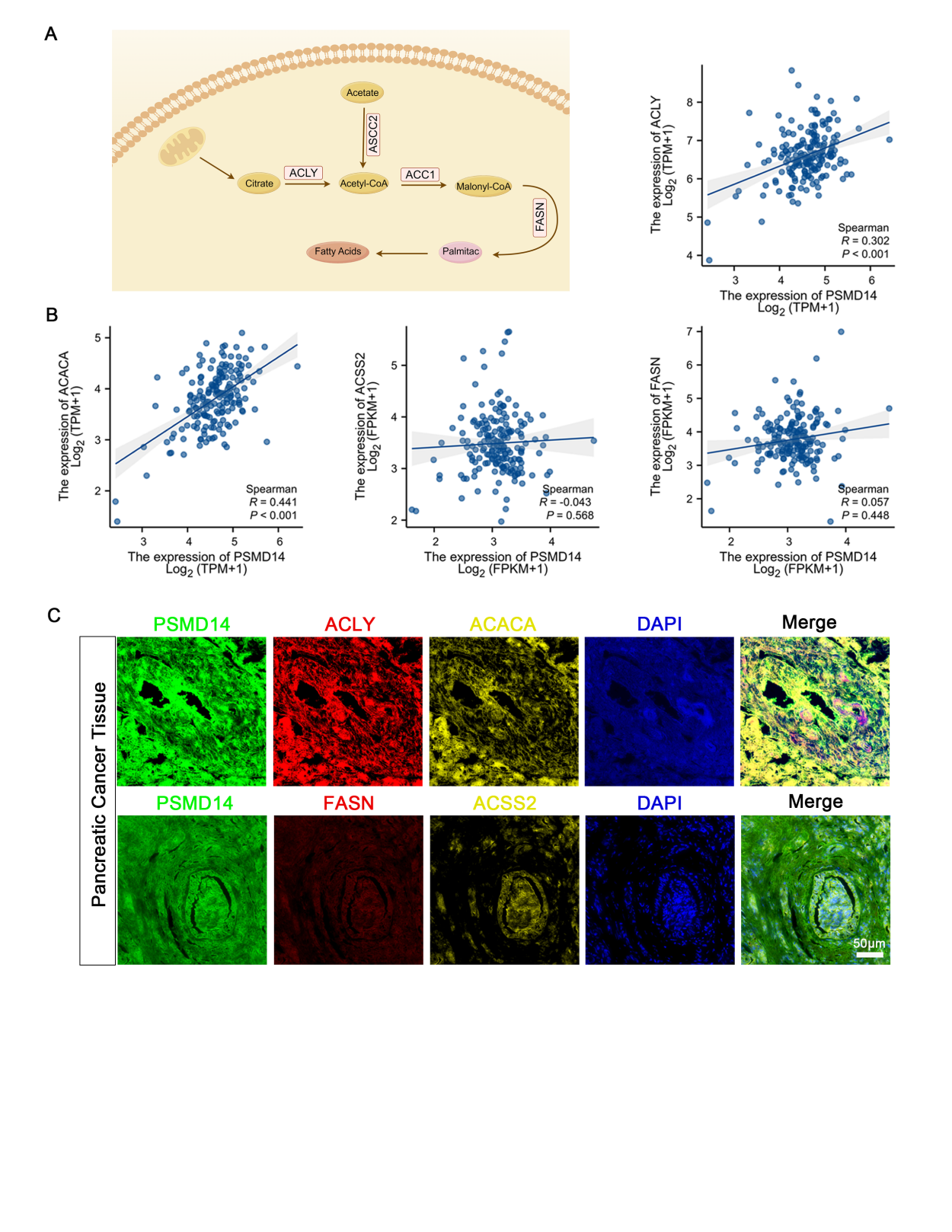
**

**Supplementary Figure S4 PSMD14 shows a positive correlation with the expression of key genes involved in fatty acid synthesis.​(A)** Schematic illustration of key components and regulatory enzyme systems within the fatty acid synthesis and metabolism pathway. **(B)** Correlation analysis revealed the relationship between PSMD14 expression and the key fatty acid synthesis genes ACLY and ACACA in TCGA data. **(C)** Immunofluorescence staining was performed to detect the co-expression of PSMD14 and fatty acid-related metabolic enzymes (ACLY, ACACA, ACSS2, and FASN) in PC tissues; n = 3 biologically independent samples.

**
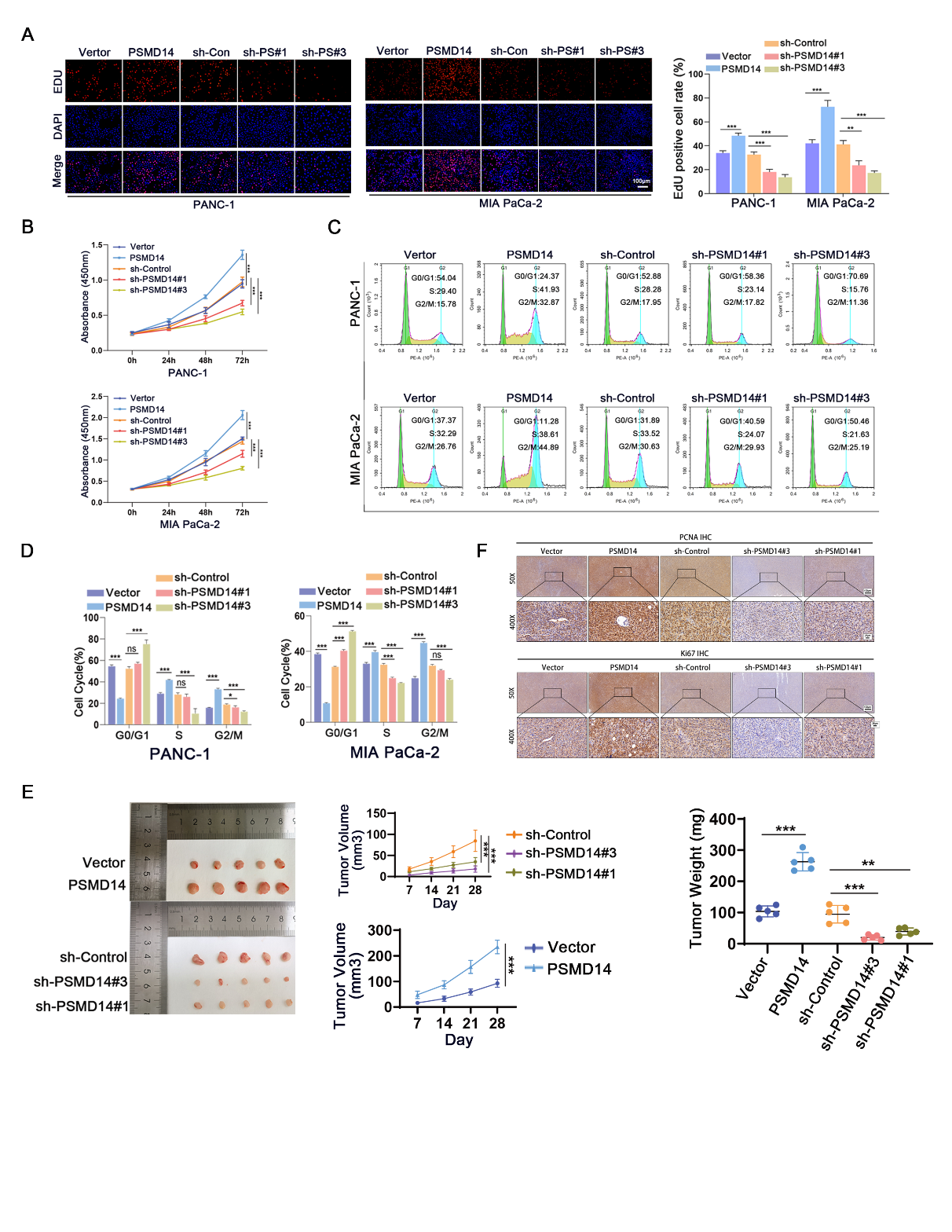
**

**Supplementary Figure S5 PSMD14 enhances the proliferation of pancreatic cancer cells both in vitro and in vivo. (A)** Representative images and statistical analysis of EdU staining for evaluating the effect of PSMD14 on PC cell proliferation; n = 3 biologically independent samples. **(B)** CCK-8 assay was performed to assess the impact of PSMD14 on PC cell proliferation; n = 3 biologically independent samples. **(C, D)** Flow cytometry analysis was conducted to determine the effect of PSMD14 on the cell cycle of PC cells, along with statistical analysis; n = 3 biologically independent samples. **(E)** Images, tumor volumes, and weights of nude mouse xenograft models established by implanting PANC-1 cells with stable overexpression or knockdown of PSMD14; n = 5 mice per group. **(F)** IHC was used to detect the expression levels of Ki67 and PCNA in tumor tissues of each group.​Data are presented as mean±SD. *p < 0.05, **p < 0.01, ***p < 0.001. P values were calculated using unpaired two-tailed Student's t-test (A, D), one-way ANOVA (A, D, E [right]), or two-way ANOVA (B, E [middle]).

**
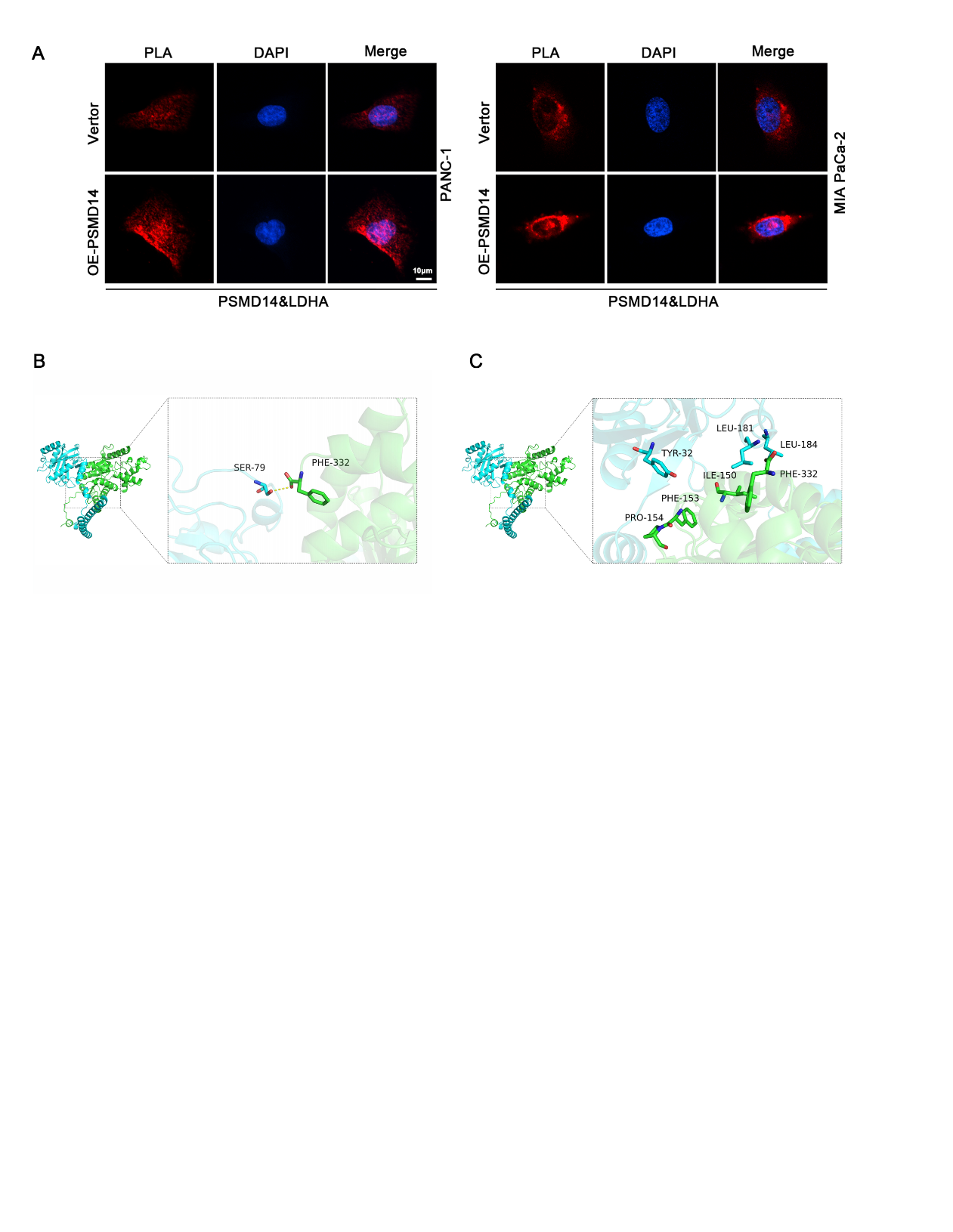
**

**Supplementary Figure S6 Prediction and verification of the interaction between PSMD14 and LDHA.​(A)** PLA fluorescent signals (red spots) demonstrate the direct binding of PSMD14 and LDHA in PANC-1 and MIA PaCa-2 cells, with cell nuclei counterstained using DAPI (blue); n = 3 biologically independent samples.

**
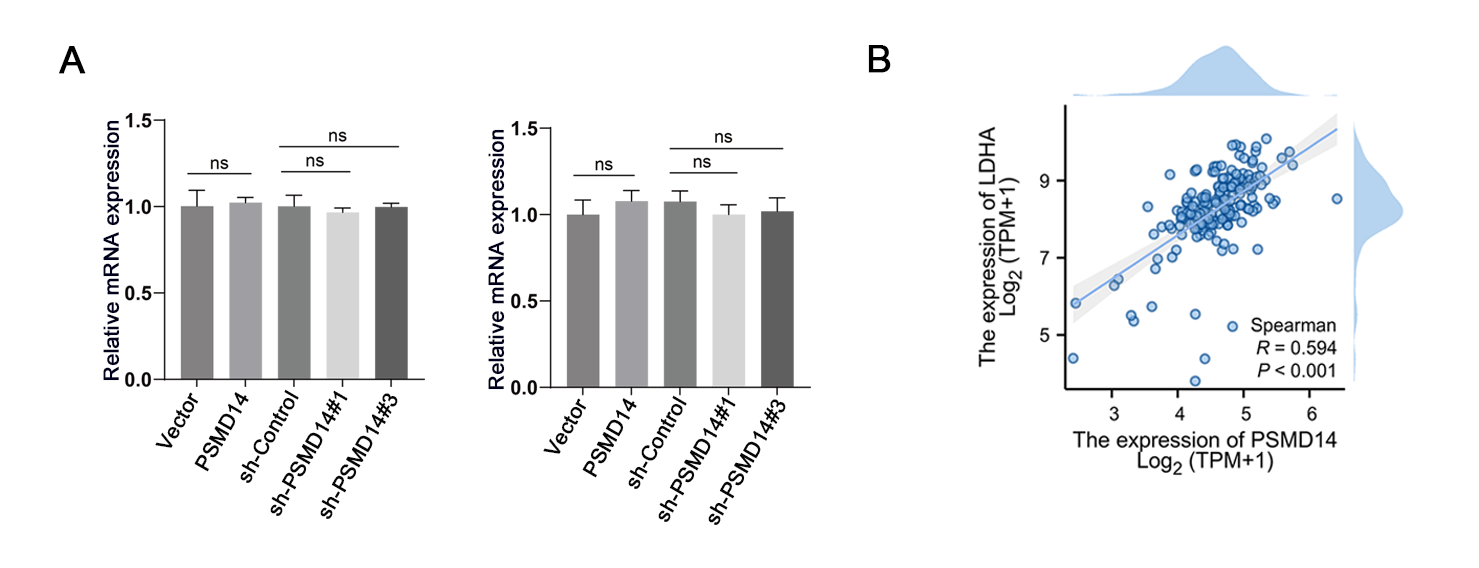
**

**Supplementary Figure S7 The regulatory effect of PSMD14 on LDHA mRNA expression. (A)** qRT-PCR was performed to examine the relationship between the expression levels of PSMD14 and LDHA in PC cells; n = 3 biologically independent samples.​Data are presented as mean±SD. **(B)** Correlation analysis revealed a relationship between PSMD14 expression and LDHA in TCGA data. *p < 0.05, **p < 0.01, ***p < 0.001. P values were calculated using unpaired two-tailed Student's t-test (A) and one-way ANOVA (A).

**
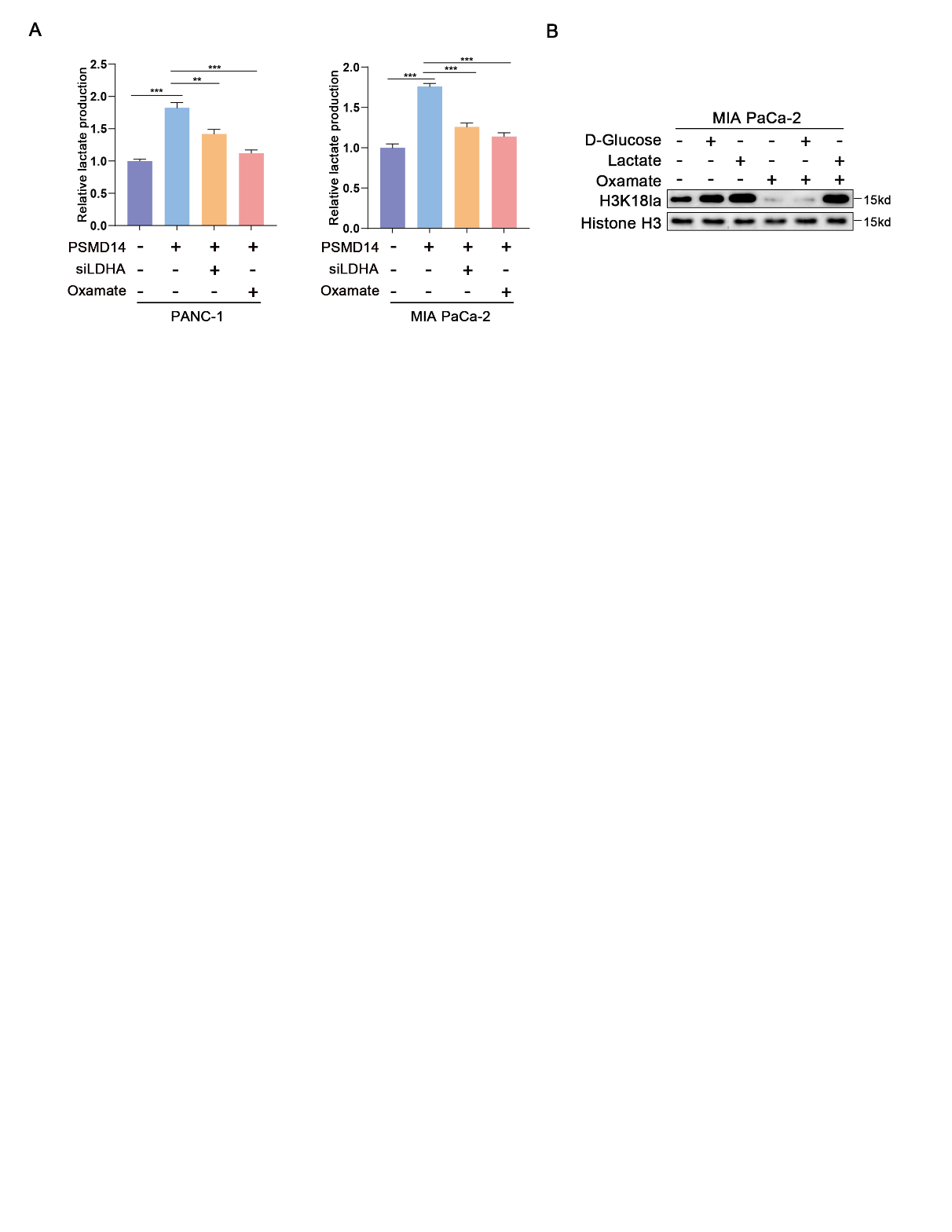
**

**Supplementary Figure S8 PSMD14 mediates lactate accumulation and histone lactylation in pancreatic cancer (PC).​(A)** In PC cells transfected with the oePSMD14 plasmid, following treatment with siLDHA or Oxamate (8 mM), lactate levels in the PC cells were analyzed using a lactate detection kit; n = 3 biologically independent samples.​Data are presented as mean±SD. *p < 0.05, **p < 0.01, ***p < 0.001. P values were calculated by one-way ANOVA (A).

**
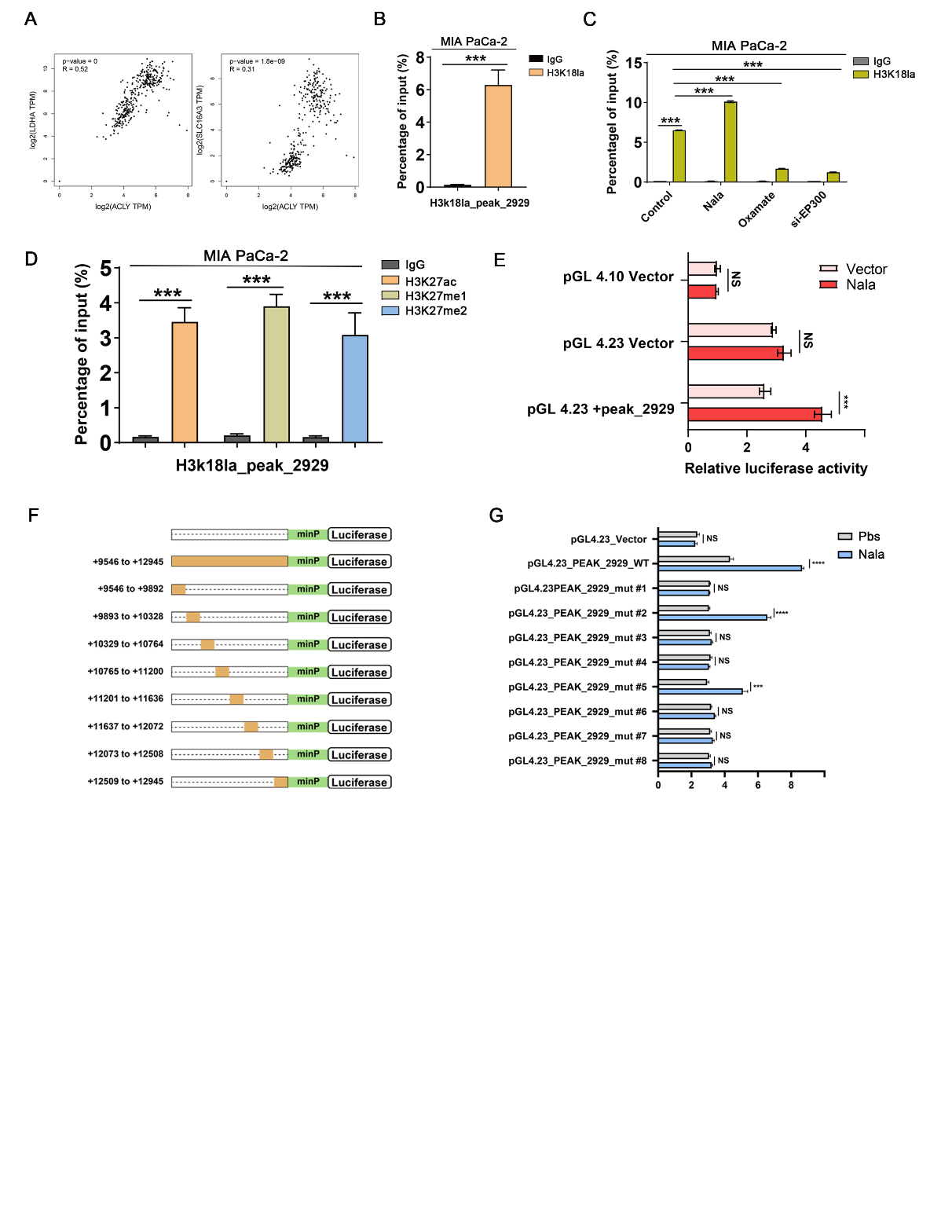
**

**Supplementary Figure S9 Histone lactylation drives ACLY gene transcription activation in pancreatic cancer.​(A)** Based on RNA-seq data from the TCGA database, correlation analysis was performed to assess the association between ACLY expression and that of LDHA or SLC16A3.​**(B)** DNA fragments from MIA PaCa-2 cells were immunoprecipitated using an H3K18la-specific antibody and analyzed via qPCR with designated primers; n = 3 biologically independent samples.​**(C)** ChIP-qPCR analysis of H3K18la status at the ACLY gene peak_2929 in MIA PaCa-2 cells treated with 20 mM Nala, 8 mM Oxamate, or transiently transfected with EP300-targeting siRNA for 24 hours, with IgG and Input serving as controls; n = 3 biologically independent samples.​**(D)** ChIP-PCR was conducted to detect the enrichment of enhancer markers H3K27ac, H3K4me1, and H3K4me2 in MIA PaCa-2 cells; n = 3 biologically independent samples. **(E)** Luciferase reporter assay was performed in MIA PaCa-2 cells transfected with different luciferase reporter vectors in the presence or absence of 20 mM Nala; n = 3 biologically independent samples.**​(F)** Schematic diagram illustrating the construction of ACLY peak_2929 wild-type (WT) and core region deletion mutant (MUT) reporter gene vectors.​**(G)** Dual-luciferase assay was carried out after transfecting wild-type (WT) or mutant vectors into PANC-1 cells and treating with 20 mM Nala for 24 hours; n = 3 biologically independent samples.​Data are presented as mean±SD. *p < 0.05, **p < 0.01, ***p < 0.001. P values were calculated using unpaired two-tailed Student's t-test (B, D, E, G) and one-way ANOVA (C).

**
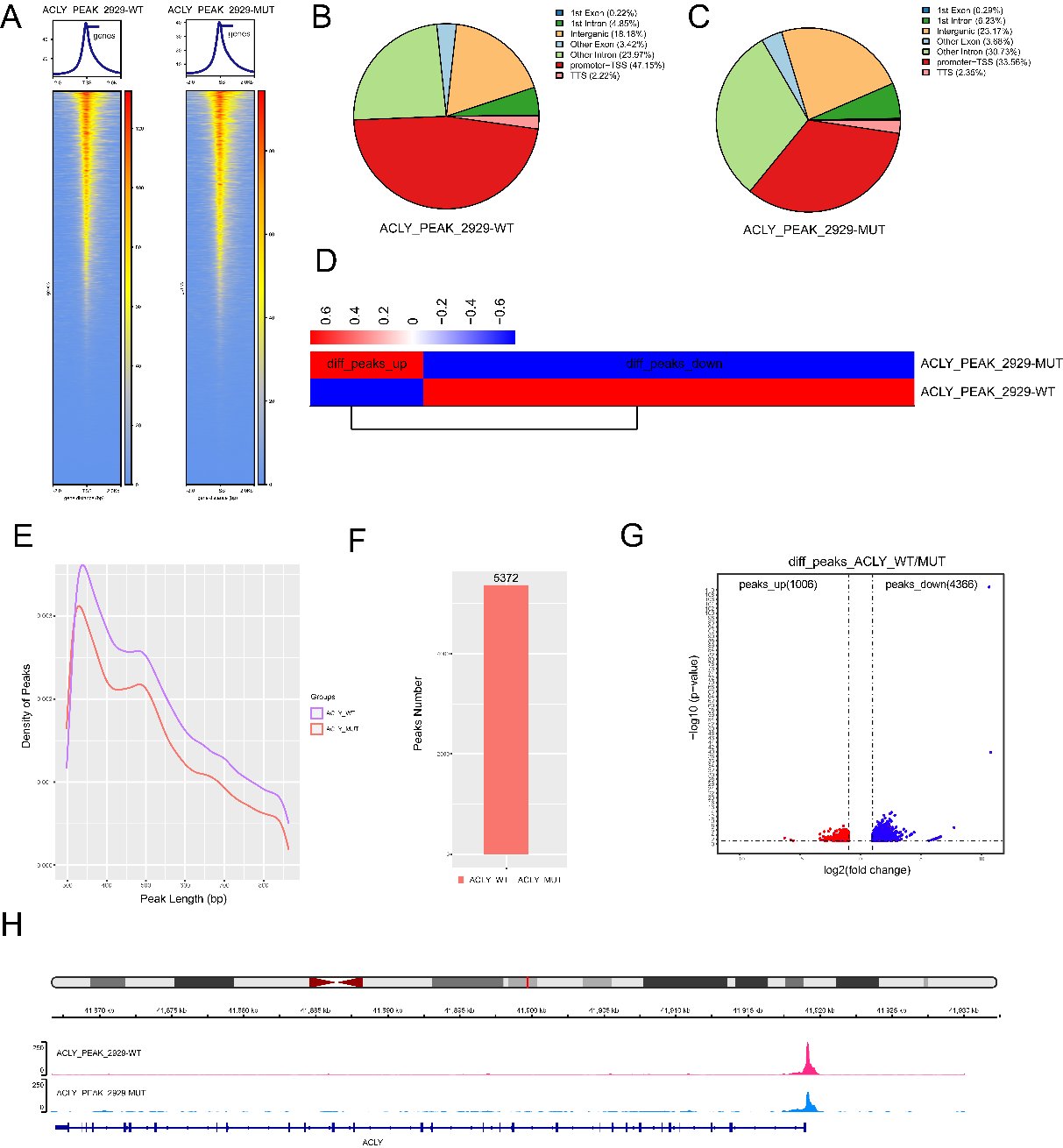
**

**Supplementary Figure S10 Effect of ACLY enhancer site mutation on chromatin accessibility. (A)** Heatmap displays read distribution near transcription start sites (TSS) in ACLY_WT versus ACLY_MUT groups. **(B-E)** Genomic distribution and variation patterns of differential peaks across groups. **(F, G)** Statistical plots showing the number and length distribution of differential peaks in each group. **(H)** Peak profile reveals reduced read density in promoter-proximal regions of the ACLY_MUT group.

**
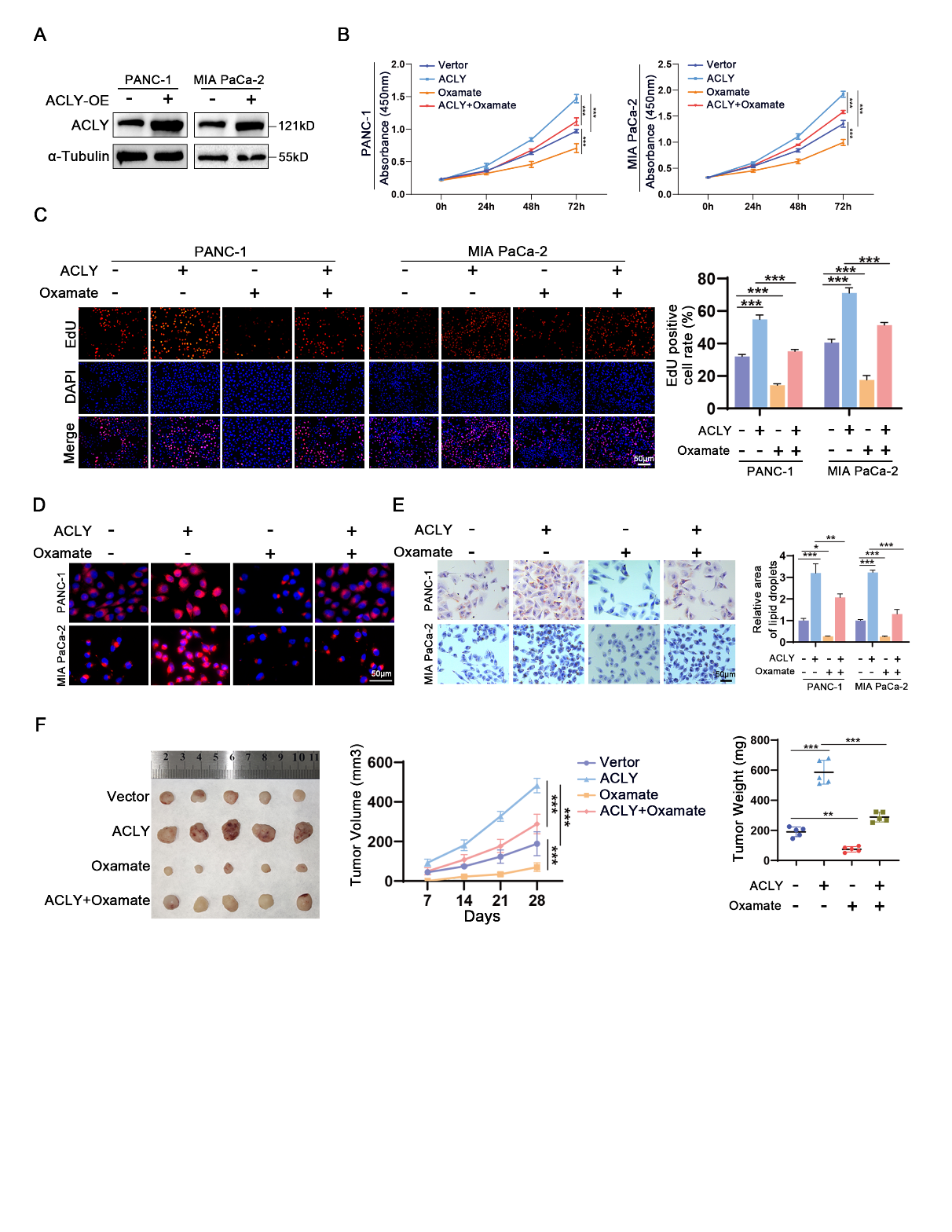
**

**Supplementary Figure S11 ACLY transcription-mediated lipid deposition is critical for pancreatic cancer (PC) cells. (A)** Western blot analysis of PANC-1 and MIA PaCa-2 cells transfected with ACLY; n = 3 biologically independent samples. **(B, C)** Proliferation of PANC-1 and MIA PaCa-2 cells with ACLY overexpression supplemented with Oxamate (8 mM) was detected by CCK-8 and EdU assays; n = 3 biologically independent samples. **(D, E)** Lipid accumulation in PANC-1 and MIA PaCa-2 cells with ACLY overexpression supplemented with Oxamate (8 mM) was determined by Nile red and ORO staining; n = 3 biologically independent samples.​**(F)** Images, tumor growth curves, and weights of subcutaneous xenografts derived from PANC-1 cells following designated treatments; n = 5 mice per group.​Data are presented as mean±SD. *p < 0.05, **p < 0.01, ***p < 0.001. P values were calculated using one-way (C, E, F [right]) or two-way (B, F [middle]) ANOVA.
