# Supplementary material for: PSMD14‐Mediated LDHA Deubiquitination Upregulates ACLY Expression via H3K18 Lactylation to Promote Lipid Synthesis and Pancreatic Cancer Progression
Source: Adv Sci (Weinh). 2025 Oct 6;12(44):e05762. doi: 10.1002/advs.202505762 (PMC12667490; doi:10.1002/advs.202505762)
Supplement: Supplementary file 2 — Supporting Information [file ADVS-12-e05762-s007.docx]

**Supplementary Table 1.** Correlation Between PSMD14 Expression and Clinicopathological Characteristics of Pancreatic Cancer Patients.

| **Clinicopathological feature** | | **PSMD14 expression, n** | | **P value** |
| --- | --- | --- | --- | --- |
|  |  | **Low expression**  **(n = 22)** | **High expression**  **(n = 38)** |  |
| **Age (years)** |  |  |  | 0.649 |
|  | <60 | 7 | 10 |  |
|  | ≥60 | 15 | 28 |  |
| **Sex** |  |  |  | 0.694 |
|  | Male | 15 | 24 |  |
|  | Female | 7 | 14 |  |
| **pTNM** **Stages** |  |  |  | **<0.001** |
|  | Ⅰ-Ⅱ | 14 | 7 |  |
|  | Ⅲ-Ⅳ | 8 | 31 |  |
| **Tumour size（cm）** |  |  |  | 0.757 |
|  | <5 | 6 | 9 |  |
|  | ≥5 | 16 | 29 |  |
| **Lymphatic node transfer** |  |  |  | 0.072 |
|  | Absent | 2 | 11 |  |
|  | Present | 20 | 27 |  |
| **Distant metastasis** |  |  |  | **0.032** |
|  | Absent | 15 | 15 |  |
|  | Present | 7 | 23 |  |
| **Number of foci** |  |  |  | 0.465 |
|  | Alone | 9 | 12 |  |
|  | Multiple | 13 | 26 |  |
| **Vascular invasion** |  |  |  | 0.464 |
|  | Absent | 12 | 17 |  |
|  | Present | 10 | 21 |  |
| **Perineural Invasion** |  |  |  | **0.024** |
|  | Absent | 18 | 20 |  |
|  | Present | 4 | 18 |  |
| **Tumour differentiation** |  |  |  | **0.037** |
|  | Poor | 10 | 25 |  |
|  | Moderate | 7 | 12 |  |
|  | Well | 5 | 1 |  |
