# Supplementary material for: PSMD14‐Mediated LDHA Deubiquitination Upregulates ACLY Expression via H3K18 Lactylation to Promote Lipid Synthesis and Pancreatic Cancer Progression
Source: Adv Sci (Weinh). 2025 Oct 6;12(44):e05762. doi: 10.1002/advs.202505762 (PMC12667490; doi:10.1002/advs.202505762)
Supplement: Supplementary file 3 — Supporting Information [file ADVS-12-e05762-s003.docx]

**Supplementary Table 2.** Cox Regression Analysis of Clinicopathological Characteristics and Prognosis in Pancreatic Cancer.

| **Clinicopathological feature** |  | **one-way analysis of variance** | | **multifactorial analysis** | |
| --- | --- | --- | --- | --- | --- |
|  |  | **HR(95% CI)** | **P** | **HR(95% CI)** | **P** |
| **Age (years)** |  |  |  |  |  |
|  | <60 |  |  |  |  |
|  | ≥60 | 0.889（0.619-1.277） | 0.524 |  |  |
| **Sex** |  |  |  |  |  |
|  | Male |  |  |  |  |
|  | Female | 0.901（0.636-1.278） | 0.561 |  |  |
| **pTNM** **Stages** |  |  |  |  |  |
|  | Ⅰ-Ⅱ |  |  |  |  |
|  | Ⅲ-Ⅳ | 0.605（0.418-0.876） | **0.008** | 0.888（0.486-1.624） | 0.700 |
| **Tumour size (cm)** |  |  |  |  |  |
|  | <5 |  |  |  |  |
|  | ≥5 | 0.943（0.643-1.382） | 0.763 |  |  |
| **Lymph Node Metastasis** |  |  |  |  |  |
|  | Absent |  |  |  |  |
|  | Present | 0.593（0.282-1.246） | 0.168 |  |  |
| **Distant Metastasis** |  |  |  |  |  |
|  |  |  |  |  |  |
|  | Present | 0.623（0.438-0.885） | **0.008** | 0.793（0.447-1.404） | 0.426 |
| **Number of Lesions** |  |  |  |  |  |
|  | Alone |  |  |  |  |
|  | Multiple | 0.837（0.583-1.202） | 0.335 |  |  |
| **Vascular Invasion** |  |  |  |  |  |
|  | Absent |  |  |  |  |
|  | Present | 0.894（0.635-1.259） | 0.521 |  |  |
| **Perineural Invasion** |  |  |  |  |  |
|  | Absent |  |  |  |  |
|  | Present | 0.557（0.378-0.821） | **0.003** | 0.846（0.556-1.289） | 0.436 |
| **Tumour differentiation** |  |  |  |  |  |
|  | Poor |  |  |  |  |
|  | Moderate | 1.909（1.157-3.151） | **0.011** | 1.404（0.796-2.476） | 0.242 |
|  | Well | 0.543（0.259-1.136） | 0.105 | 0.614（0.274-1.378） | 0.237 |
| **PSMD14** |  |  |  |  |  |
|  | Low |  |  |  |  |
|  | High | 0.361（0.222-0.589） | **<0.001** | 0.419（0.245-0.717） | **0.001** |
