# Supplementary material for: PSMD14‐Mediated LDHA Deubiquitination Upregulates ACLY Expression via H3K18 Lactylation to Promote Lipid Synthesis and Pancreatic Cancer Progression
Source: Adv Sci (Weinh). 2025 Oct 6;12(44):e05762. doi: 10.1002/advs.202505762 (PMC12667490; doi:10.1002/advs.202505762)
Supplement: Supplementary file 4 — Supporting Information [file ADVS-12-e05762-s001.docx]

**Supplementary Table 3. s**iRNA sequences used in our experiement.

| **Gene** | **Targeting sequences** |
| --- | --- |
| siPSMD14#1 | 5′-AAUGCCUGGAUAGAUGGCUUGTT-3′ |
| siPSMD14#2 | 5′-UUGUGUUUACAAUGUUCACUGTT-3′ |
| siPSMD14#3 | 5′-UAUCCACACCAGAAAGCCAACTT-3′ |
| siLHDA | 5′- GAGGTCCTCTGCATGGATT-3′ |

shRNA sequences used in our experiement.

| NO. | 5’ | STEM | Loop | STEM | 3’ |
| --- | --- | --- | --- | --- | --- |
| sh-PSMD14#1-a | Ccgg | AAUGCCUGGAUAGAUGGCUUGTT | CTCGAG | ATCAAGCCATCTATCCAGGCATT | TTTTTg |
| sh-PSMD14#1-b | aattcaaaaa | AAUGCCUGGAUAGAUGGCUUGTT | CTCGAG | ATCAAGCCATCTATCCAGGCATT |  |
| sh-PSMD14#3-a | Ccgg | UAUCCACACCAGAAAGCCAACTT | CTCGAG | AAGTTGGCTTTCTGGTGTGGAUA | TTTTTg |
| sh-PSMD14#3-b | aattcaaaaa | UAUCCACACCAGAAAGCCAACTT | CTCGAG | AAGTTGGCTTTCTGGTGTGGAUA |  |
| sh-LHDA-a | Ccgg | GAGGTCCTCTGCATGGATT | CTCGAG | AATCCATGCAGAGGACCTC | TTTTTg |
| sh-LHDA-b | aattcaaaaa | GAGGTCCTCTGCATGGATT | CTCGAG | AATCCATGCAGAGGACCTC |  |
