# Supplementary material for: PSMD14‐Mediated LDHA Deubiquitination Upregulates ACLY Expression via H3K18 Lactylation to Promote Lipid Synthesis and Pancreatic Cancer Progression
Source: Adv Sci (Weinh). 2025 Oct 6;12(44):e05762. doi: 10.1002/advs.202505762 (PMC12667490; doi:10.1002/advs.202505762)
Supplement: Supplementary file 5 — Supporting Information [file ADVS-12-e05762-s005.docx]

**Supplementary Table 4.** Primers used in this study.

**RT-qPCR primers:**

| **Gene** | **Sequences (5’-3’)** |
| --- | --- |
| PSMD14-Forward | TTTGCTATGCCACAGTCAGGA |
| PSMD14-Reverse | AGCCAGGGTGACTGTGATAC |
| LDHA-Forward | ATGGCAACTCTAAAGGATCAGC |
| LDHA-Reverse | CCAACCCCAACAACTGTAATCT |
| ACLY-Forward | TCGGCCAAGGCAATTTCAGAG |
| ACLY-Reverse | CGAGCATACTTGAACCGATTCT |
| α-Tubulin-Forward | ACCAACCTGGTGCCCTATCC |
| α-Tubulin-Reverse | CAAGCATTGGTGATCT |

**ChIP-qPCR primers:**

| **Gene** | **Sequences (5’-3’)** |
| --- | --- |
| ACLY-Forward | CGCTGGAATCTCGCATTGAA |
| ACLY-Reverse | CAGTGTGGGATAAGGCAGGG |
