# Supplementary figures and images for: PSMD14‐Mediated LDHA Deubiquitination Upregulates ACLY Expression via H3K18 Lactylation to Promote Lipid Synthesis and Pancreatic Cancer Progression
Source: Adv Sci (Weinh). 2025 Oct 6;12(44):e05762. doi: 10.1002/advs.202505762 (PMC12667490; doi:10.1002/advs.202505762)

Supplemental Material – Original Blots


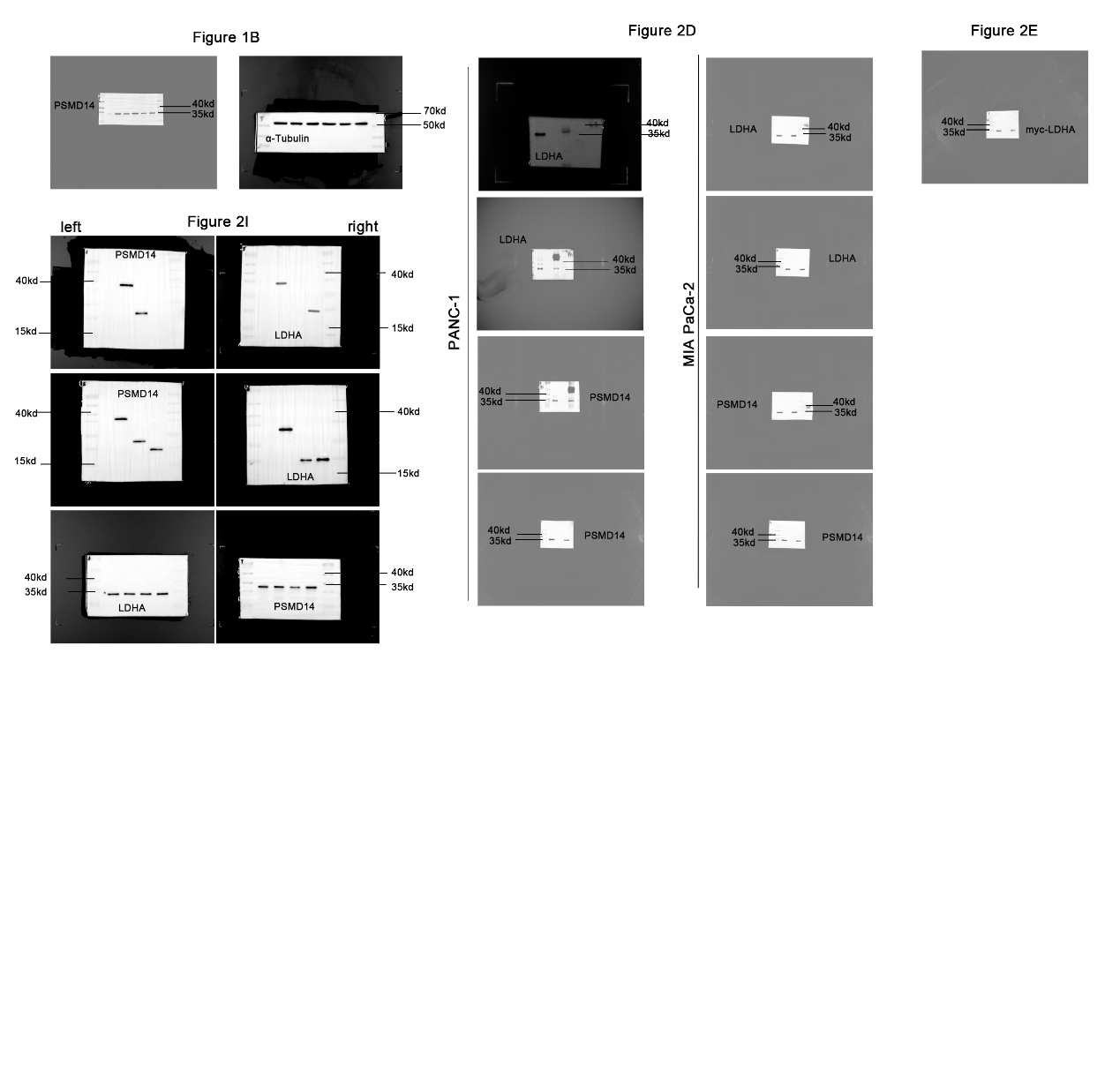


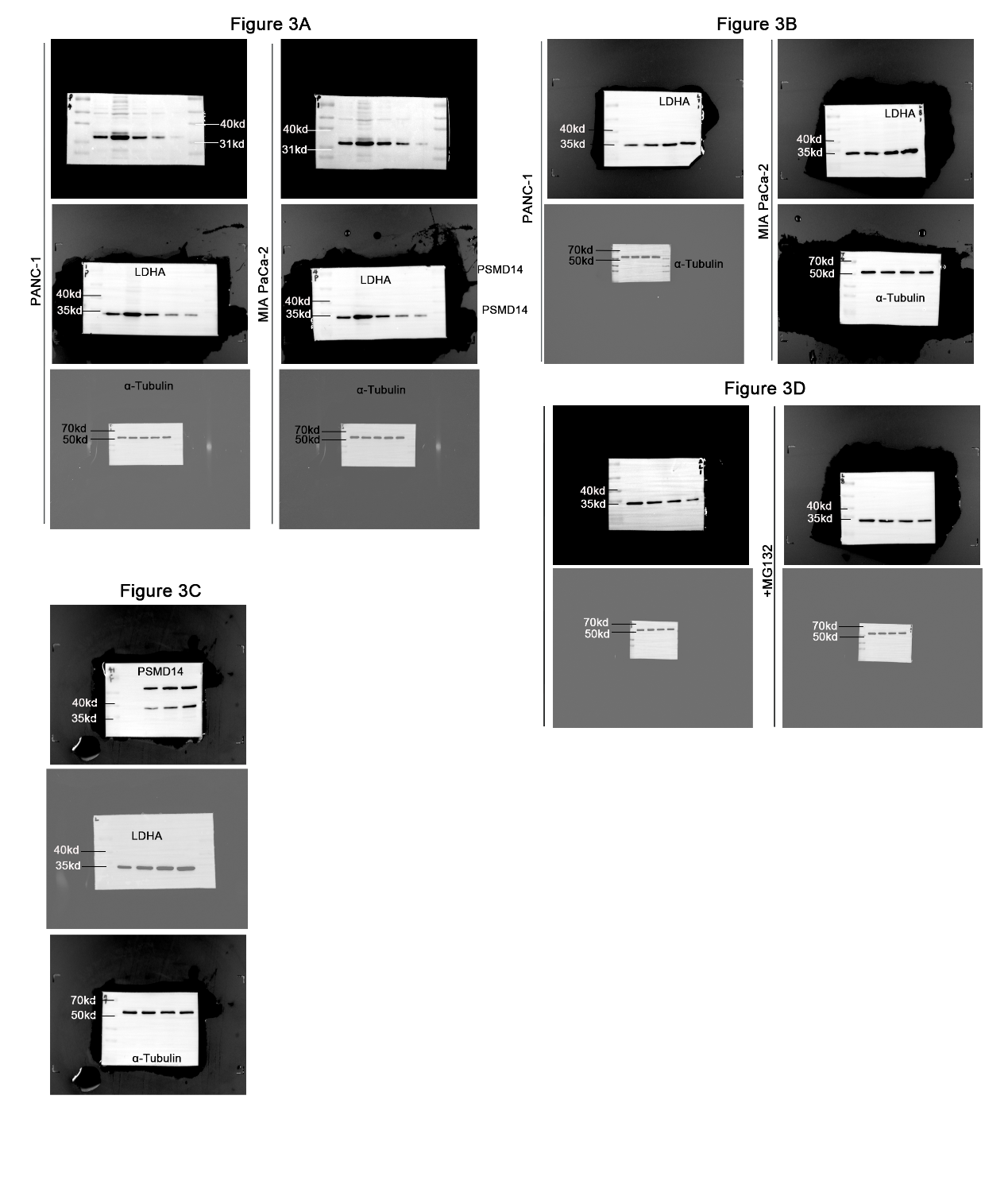


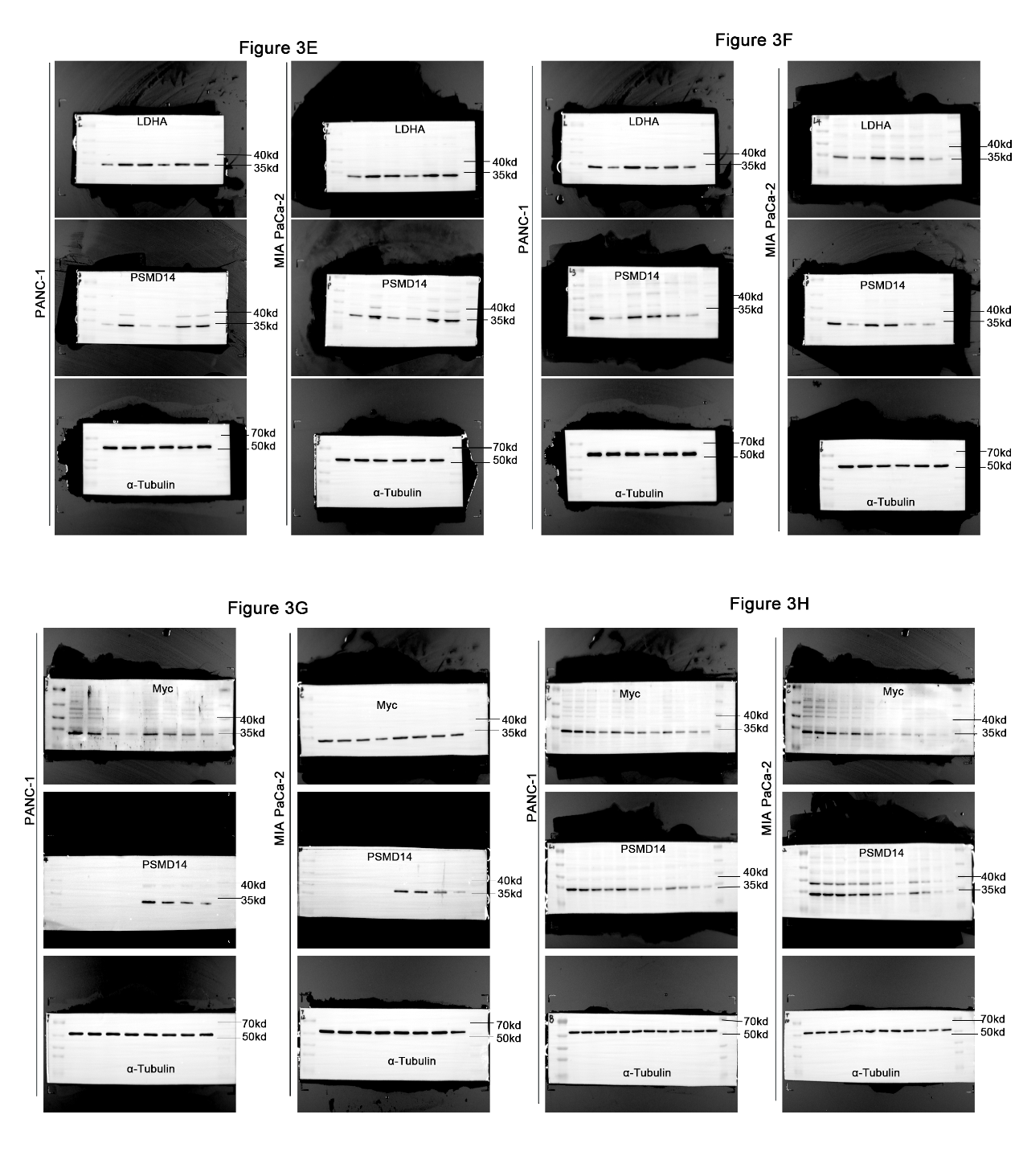


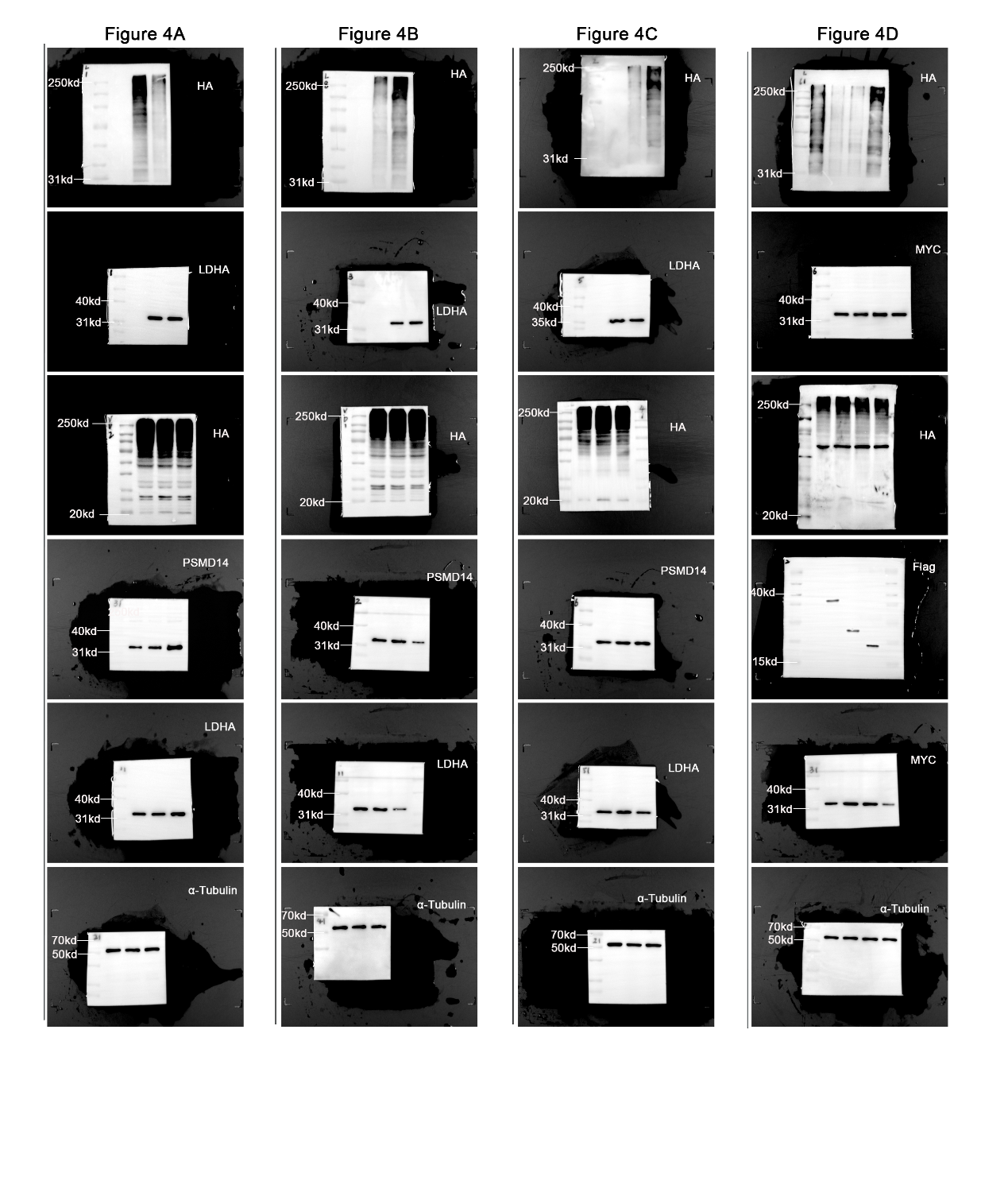


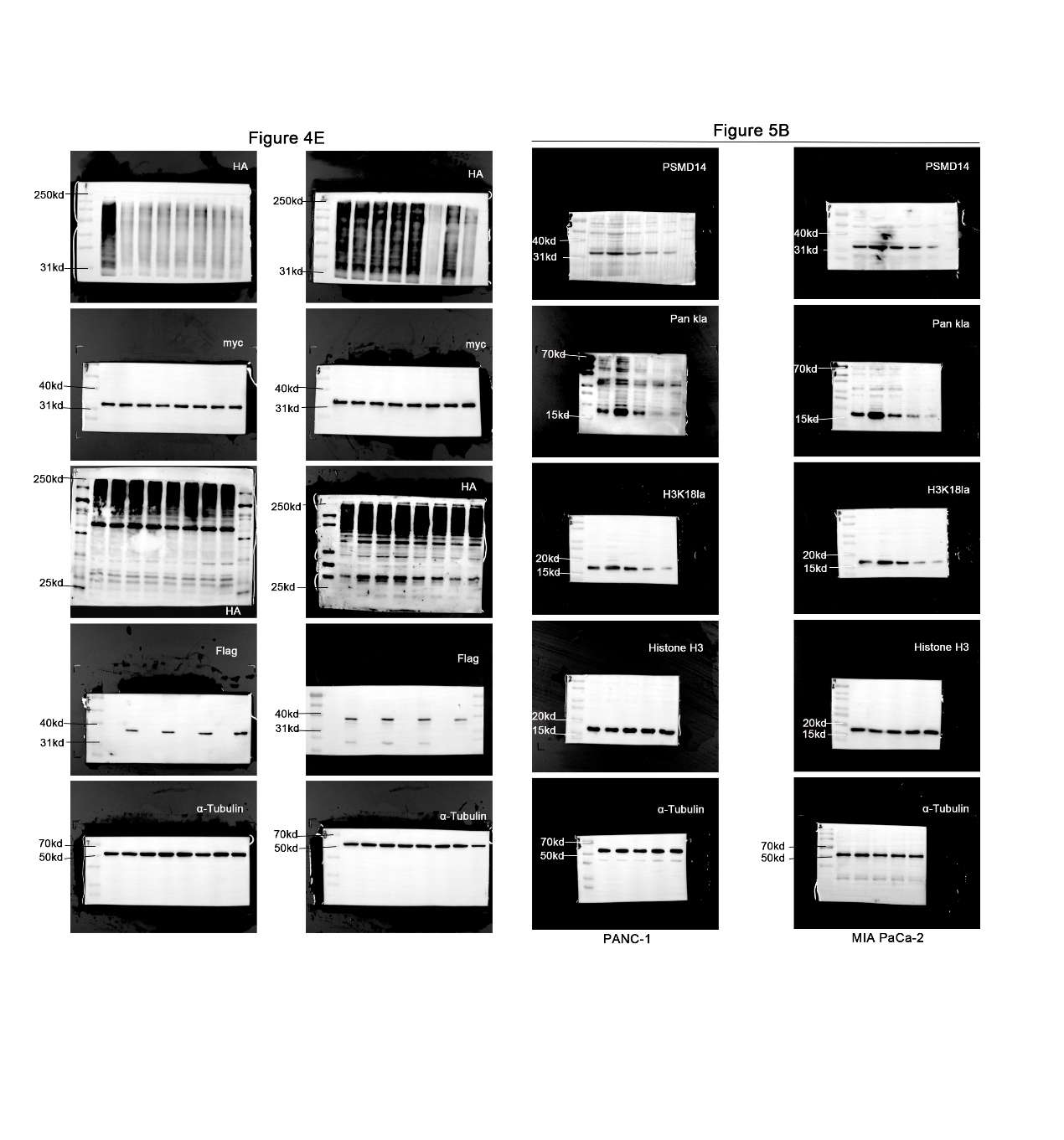


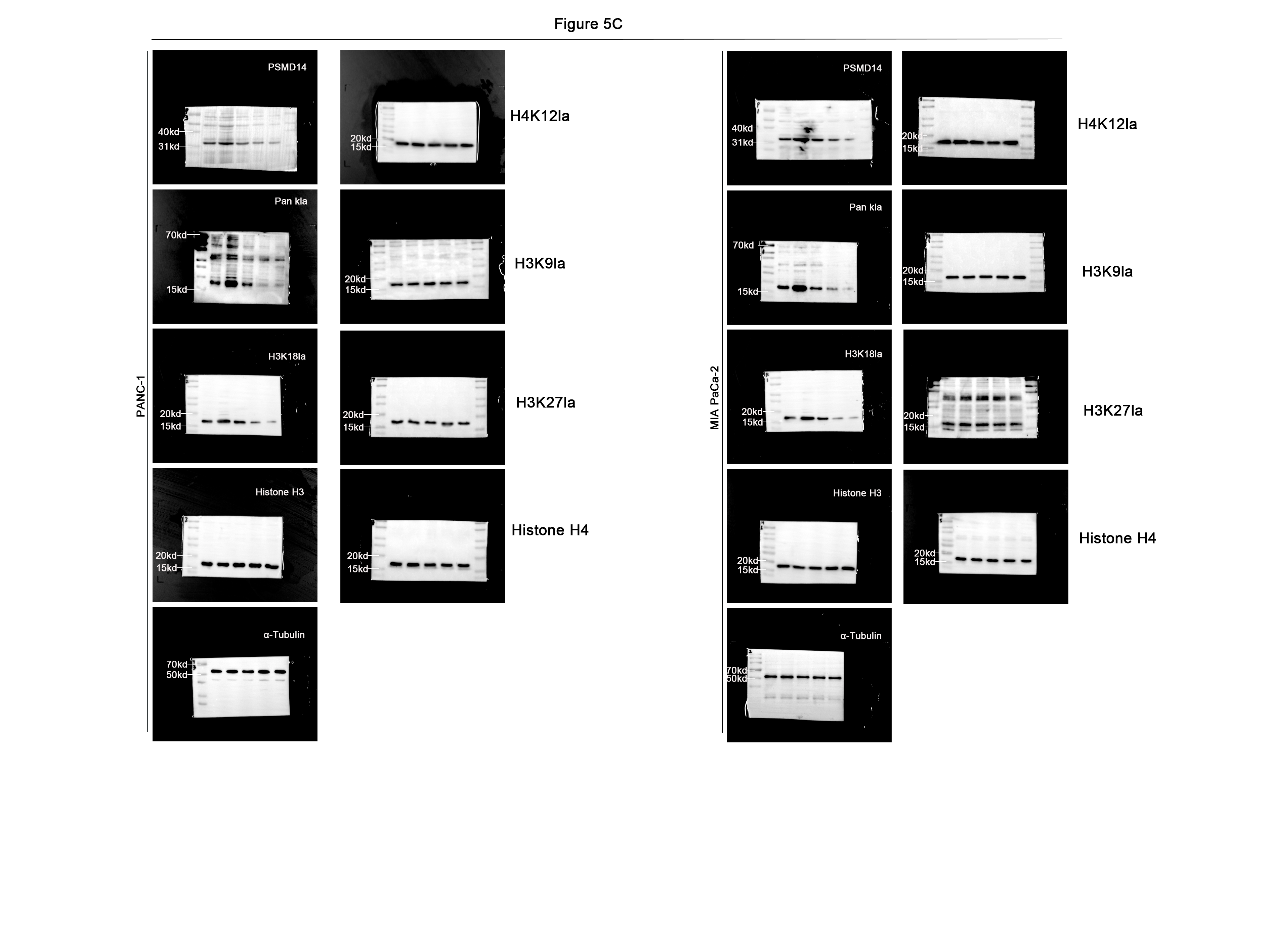


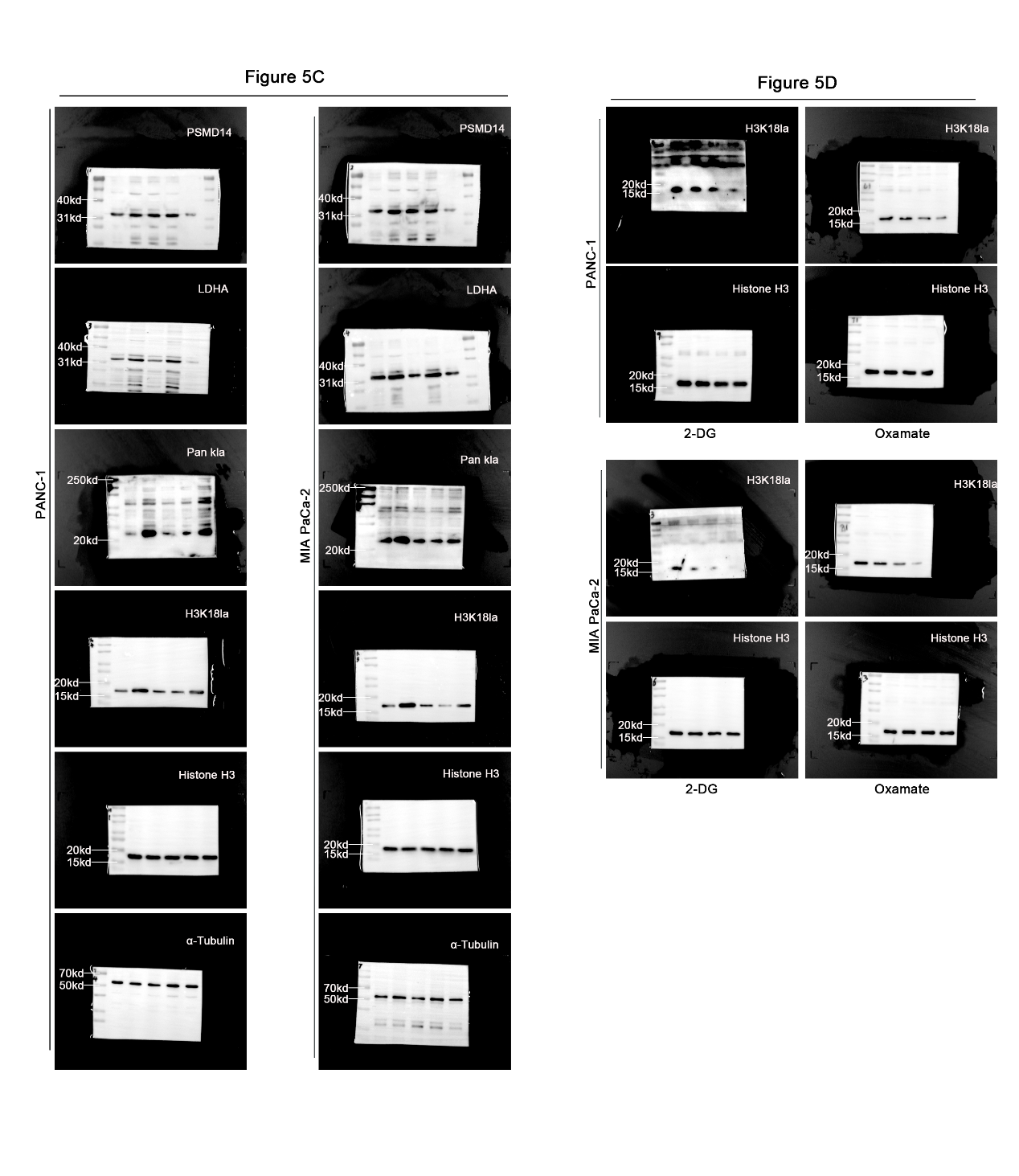


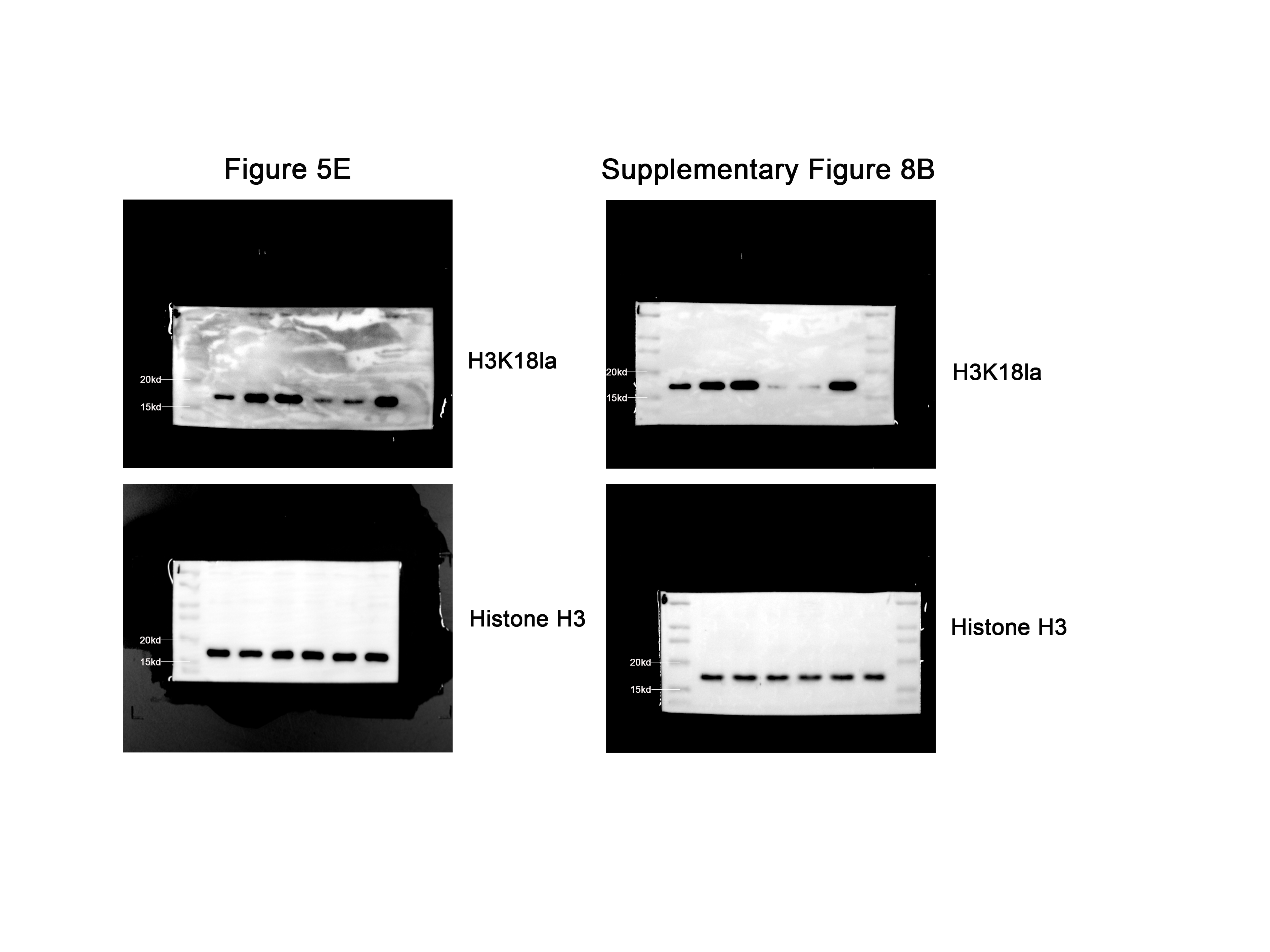


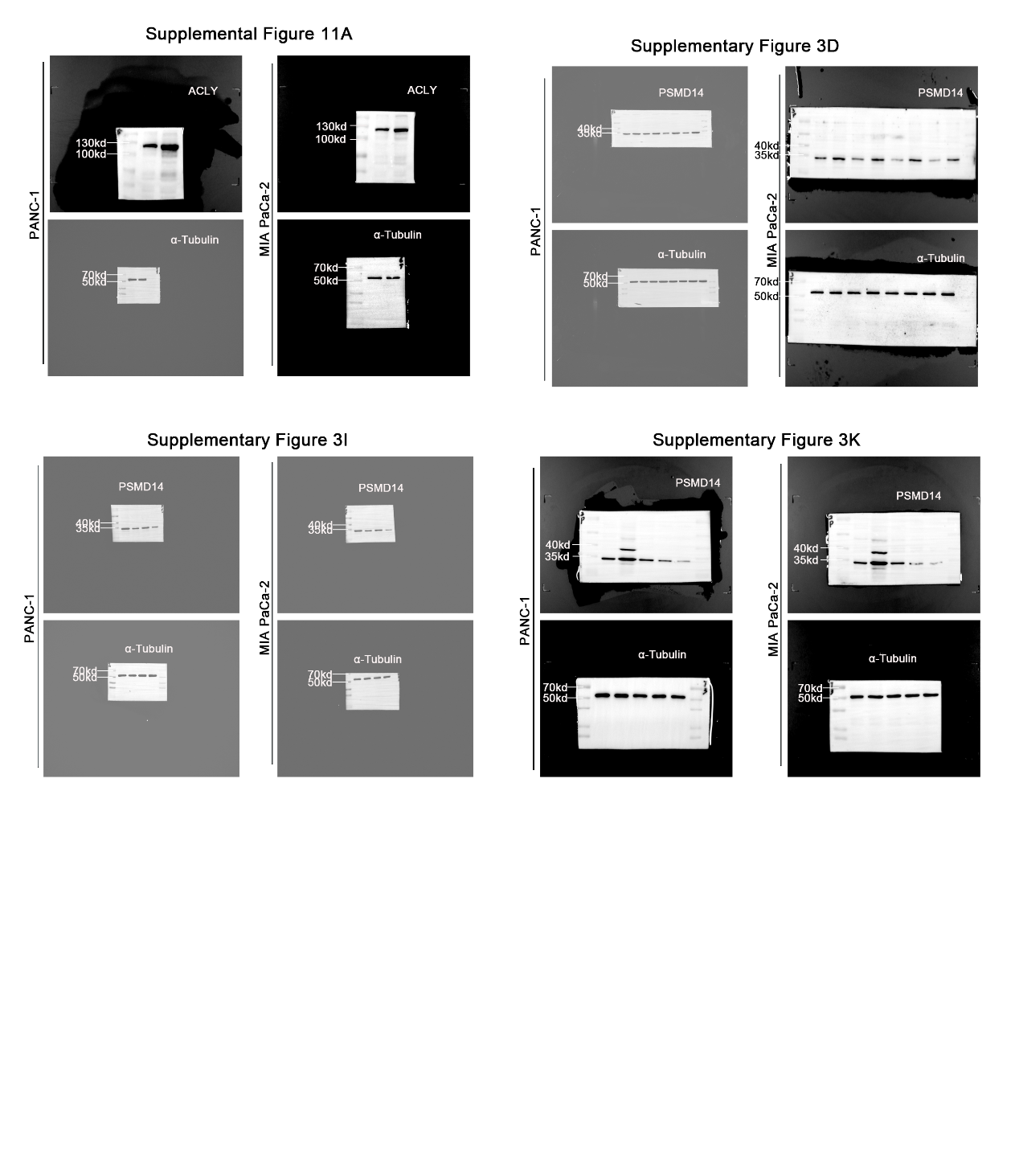


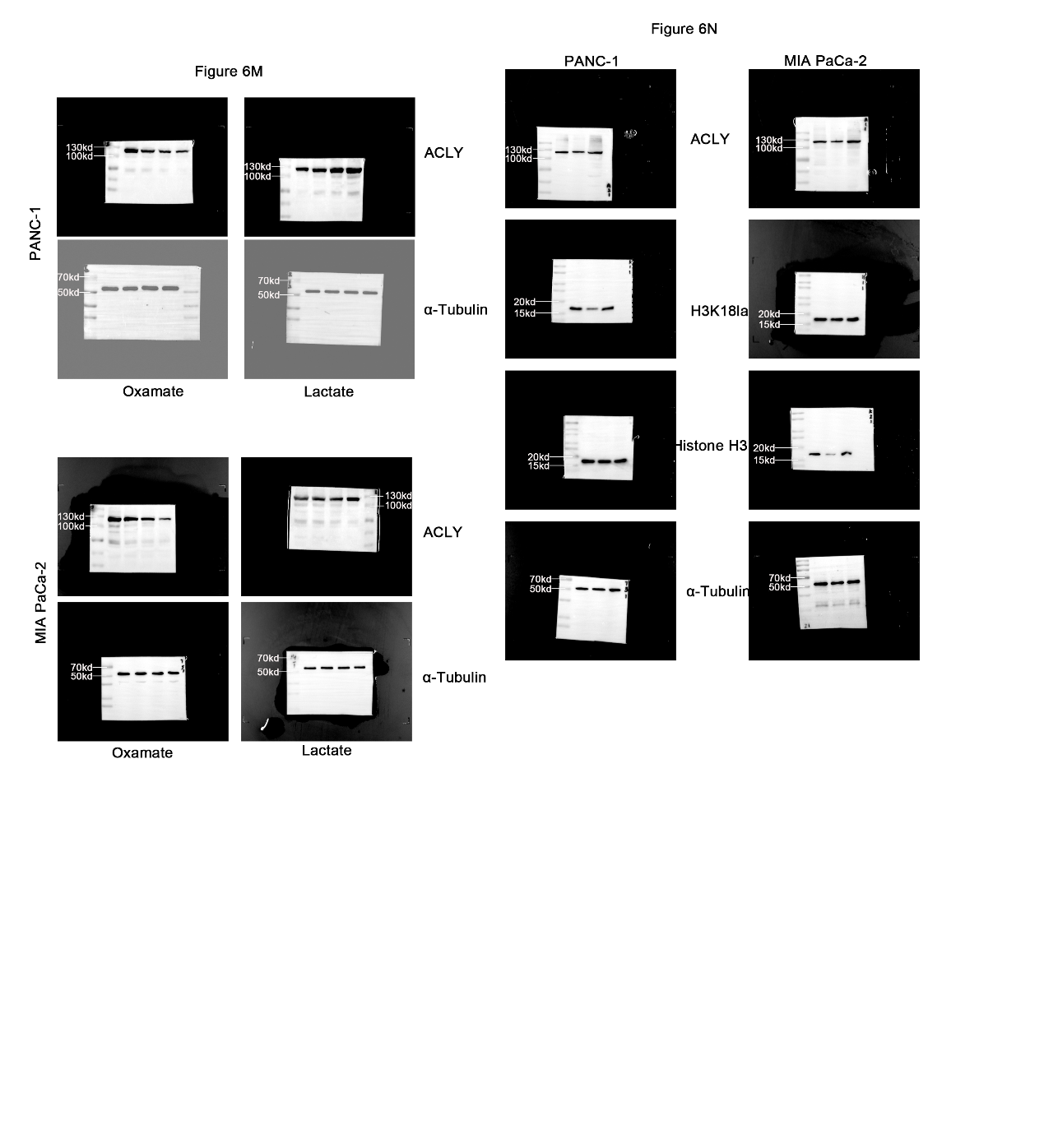

Supplement: Supplementary file 7 — Supporting Information [file ADVS-12-e05762-s004.docx]
